# Roles of ribosomal RNA in health and disease

**DOI:** 10.3389/frnar.2023.1331185

**Published:** 2024-01-18

**Authors:** Ryan Johnston, Anne Aldrich, Shawn M. Lyons

**Affiliations:** 1 Department of Biochemistry and Cell Biology, Chobanian and Avedisian School of Medicine, Boston, MA, United States,; 2 Genome Science Institute, Chobanian and Avedisian School of Medicine, Boston, MA, United States

**Keywords:** ribosome, translation, rRNA, ribosomopathy, protein synthesis

## Abstract

Ribosomes are amongst the most ancient molecular machines in cells, showing conservation from the simplest prokaryotes to humans. Ribosomes are an assembly of ribosomal (r)RNA and ribosomal proteins, but the rRNA comprises most of the mass of the ribosome and performs key enzymatic tasks. In humans, rRNA undergoes a laborious maturation that involves multiple processing steps and the deposition of chemical modifications. The correct processing and modification of rRNA ensures the proper function of the mature ribosome. Disturbance of these processes may lead to human disease. Understanding the role of rRNA in protein synthesis and the consequences of its dysregulation is key to deciphering and mitigating the emergence of pathological states in human biology.

## Introduction

*De novo* protein synthesis is a requirement of all forms of life. Cellular proliferation increases the demand for new proteins. Ribosomes are the macromolecular machines tasked with synthesizing new proteins; ironically, their synthesis represents a major source of new protein production. Ribosome biogenesis accounts for more than 60% of energy consumption in a growing cell (Warner et al., 2001) due to both their complexity and size.

The human 80S ribosome is 4.3 megadaltons and is composed of 80 ribosomal proteins and 4 non-coding RNAs. Owing to the centrality of protein synthesis in life, both ribosomes and tRNAs are conserved throughout evolution, despite the stark differences in translation regulation—particularly at the initiation stage. Some 33 of the 80 human ribosomal proteins are conserved in *Escherichia coli*, which diverged from humans 3–4 billion years ago. Bacteria have 55, rather than 80, ribosomal proteins, meaning that 60% of their ribosomal proteins are conserved in humans (Table 1).

From prokaryotes to eukaryotes, rRNA plays a disproportionate role in the activity of ribosomes. rRNA coordinates accessory factor binding, plays a key role in discriminating amongst incoming charged tRNAs to ensure proper mRNA decoding, and, perhaps most importantly, rRNA plays a critical role in catalyzing peptide bond synthesis. This role increases the rate of peptide bond formation by a factor of 2 × 10^7^ (Sievers et al., 2004). Emerging evidence also suggests that rRNA is a source of heterogeneity amongst ribosomes and hints at specialized ribosomes.

In this review, we will discuss the maturation of rRNA and its role in protein synthesis. We will also highlight disorders that arise from defects in these processes, with an emphasis on human disease.

## Functions of rRNA in canonical translation

To better understand how ribosome malfunctions related to rRNA contribute to disease, it is first necessary to understand the canonical roles that rRNA plays in protein synthesis. Following translation initiation, the joined 80S ribosome translocates along an mRNA, catalyzing new polypeptides as it goes. The large 60S subunit contains three sites through which tRNAs and mRNAs traverse to facilitate this activity: the aminoacyl (A), peptidyl (P), and exit (E) sites. During translocation, an individual tRNA will move from the A to the P site, from the P to the E site, or from the E site out of the ribosome. The A site contains aminoacylated tRNAs still conjugated to their cognate amino acid, the P site contains a peptidyl tRNA conjugated to the growing polypeptide chain, and the E site contains a deacylated tRNA ready for expulsion from the ribosome. As they enter, the tRNA and mRNA complex first encounters the decoding center (DC) before being transferred to the catalytic site, known as the peptidyl transferase center (PTC). The solution of the crystal structures of the large subunits, initially from prokaryotes and later in eukaryotes, reveals that the functional core of the PTC is rRNA and that rRNA plays a critical role in interrogating and proofreading tRNAs in the DC.

Many of the mechanistic studies regarding the molecular interactions of mRNA, tRNA, and the ribosome were initially conducted in prokaryotic systems. Subsequent data have revealed key similarities and distinctions between prokaryotic and eukaryotic ribosomes. An aminoacyl (aa)-tRNA is transported to the ribosome by EF-Tu, the bacterial homolog of mammalian eEF1a, where it is sampled at the DC. The ribosome must function as a “gatekeeper” in which non-cognate tRNAs are distinguished from cognate tRNAs. This is initially monitored by base-pairing between the mRNA codon and the anti-codon of the tRNA, although this is not sufficient for codon recognition.

Pioneering work by the Noller Lab showed that chemical modification of the 16S rRNA—the bacterial homolog of mammalian 18S—by kethoxal abolished tRNA binding to the A site of the ribosome (Noller and Chaires, 1972). Further chemical probing used bound tRNAs to protect against chemical modification and identified specific nucleotides of the 16S rRNA that mediated tRNA binding: G_530,_ A_1492_, and A_1493_ (Moazed and Noller, 1990). Homologous nucleotides in the 18S rRNA of humans—G_626,_ A_1824_, and A_1825_—serve similar roles (Table 2). When a cognate tRNA binds to a codon through its anticodon, this interaction is validated by flipping of A1492/1824 and A1493/1825, and conversion of G530/626 from a *syn-* to an *anti*-conformation. This facilitates an “A-minor interaction” with the first two base pairs in the codon–anticodon helix (Ogle et al., 2001). The mutational analysis further demonstrates the essential nature of these universally conserved bases (Cochella et al., 2007). This triggers large-scale movements of the small ribosome subunit (Ogle et al., 2002). In this conformation, there is evidence in prokaryotes that the 23S rRNA plays some role in stabilizing cognate tRNAs within the A site. A_1913_ of the 23S rRNA interacts with the A site tRNA within the DC directly and via Mg^2+^-mediated contacts (Selmer et al., 2006). Mammalian ribosomes similarly interact with tRNAs (Lomakin and Steitz, 2013). These movements prime EF-Tu/eEF1a for GTP hydrolysis to complete the first step of tRNA selection. In both prokaryotes and eukaryotes, EF-Tu/eEF1a bind the sarcin–ricin loop (SRL) of the 23S/28S rRNA, indicating the role of rRNA in the recruitment of elongation factors during translation (Correll et al., 1998; Lancaster et al., 2008; Budkevich et al., 2014). In prokaryotes, A_2662_ of the 23S rRNA interacts with His-84 of EF-Tu to stimulate GTP hydrolysis and, ultimately, EF-Tu dissociation. In eukaryotes, this mechanism is coordinated by the interaction of A_4607_ of the 28S rRNA and catalytic His-95 of eEF1a (Shao et al., 2016). At this point, interactions within the DC between rRNA and tRNA are a major driving force in cognate tRNA stability within the ribosome. Thermodynamic properties make non-cognate tRNAs more likely to disassociate at this point. The conformational changes made by cognate tRNA recognition instead drive the tRNA toward the P site and the PTC. Recent data demonstrate that the decoding mechanism in humans is ten times slower than in prokaryotes and may be functionally distinct (Holm et al., 2023). Despite the great strides gained from the study of the prokaryotic system, these results highlight the need for additional analysis of the mechanism of human ribosomal function.

The role of rRNA within the PTC and its role in catalyzing peptide bond synthesis cannot be overstated (Steitz and Moore, 2003). Ultimately, the mechanism of peptide bond formation was validated by crystallographic analysis of ribosomes; the first evidence that rRNA, rather than proteins, was responsible for catalysis was gained through biochemical analysis.

In hindsight, *in vitro* reconstitution experiments using *E. coli* components initially provided the first evidence that rRNA played a key role in peptidyl transferase activity. The 23S rRNA was identified as one of the essential factors required for reconstituting peptidyl transferase activity *in vitro* (Hampl et al., 1981; Schulze and Nierhaus, 1982). However, these studies also identified six ribosomal proteins that were essential, so activity could not be attributed to the rRNA.

The prokaryotic 23S rRNA has six major domains (Noller et al., 1981). Due to its increased photoreactivity when irradiated with 320 nm light and crosslinking to both RNA and protein, 3-(4′-benzoylphenyl)propionyl (BP)-[^3^H]-tRNA^Phe^ was used as a chemical probe. The tRNAs crosslinked directly to the central single-stranded loop of domain V of the 23S rRNA in the PTC (Barta et al., 1984; Steiner et al., 1988). Primer extension determined that the tRNA crosslinked to U_2584_ and U_2525_ in the A site and to A_2451_ and C_2452_ in the P site—early evidence that RNA, not protein, was at the catalytic heart of the ribosome.

Chemical probing of tRNA-bound 70S ribosomes also showed that tRNAs bound in the P site would protect the PTC from chemical modification (Moazed and Noller, 1986). The protective effect was contingent upon the tRNA’s “CCA end”. This is critical because the CCA end is the site of amino acid conjugation, suggesting the 23S rRNA is near the site of catalysis. Antibiotic studies also provided key insights into the role of rRNA in peptide bond formation, as chemical probing showed that several antibiotics which were known to inhibit peptidyl transferase activity would protect nucleotides near A_2451_ (Moazed and Noller, 1987). These studies suggested that the antibiotics target the same region of 23S rRNA, in domain V, to block protein synthesis. Strong biochemical evidence was also presented by demonstrating that purified ribosomes treated with proteinase K, or partially extracting proteins, did not abolish peptidyl transferase activity. However, treatment with RNase T1 was sufficient to inhibit this activity (Noller et al., 1992).

While these experiments provided evidence that peptide bond formation was an rRNA-catalyzed event, the solution of the crystal structure of the 50S ribosome gave the clearest evidence that this was so (Ban et al., 2000; Nissen et al., 2000). This structure from the archaeon *Haloarcula marismortui* showed that A_2451_ was positioned precisely to mediate peptide bond synthesis. This suggested a model through which the *N*^3^ of A_2451_ could act to catalyze the reaction through general acid-base catalysis (Nissen et al., 2000). Later work refined this model to show that the 2′-OH of this nucleotide was the critical functional group (Erlacher et al., 2005). If A_2451_ were replaced with deoxyA_2541_, catalysis was severely compromised. Follow-up work demonstrated that the 2′-OH group of A_2541_ could act as a hydrogen donor during catalysis (Lang et al., 2008). This model was further refined using a higher resolution structure of the ribosome that revealed an additional water molecule present in the PTC (Polikanov et al., 2014). It has been proposed that this water molecule acts as a proton wire between the 2′-OH group of A_2451_ and A_76_ of the tRNA.

Finally, regions of rRNA beyond the core catalytic domains have been implicated in ribosome function. As the rRNA lengths have increased throughout evolution, much of the added length can be found in the aptly named “expansion segments (ES).” Analysis of some of the first structures of eukaryotic ribosomes revealed that they play many important roles. Most notably, these extensions form many eukaryotic-specific inter-subunit bridges between the 60S and 40S ribosomes (Ben-Shem et al., 2011). Expansion segments have also been shown to be directly involved in protein synthesis. In yeast, ES27Lb coordinates an interaction with NatA—a complex of proteins that acetylates the N-terminus of nascent peptides (Knorr et al., 2019). Binding at this ES positions NatA at the peptide exit tunnel so that it is in the opportune position to acetylate newly synthesized proteins. Deletion of ES27Lb sensitizes cells to reductive stresses by causing an increase in protein aggregates (Shankar et al., 2020). While ΔES27Lb cells show no difference in global translation levels, there are transcript-specific defects. This highlights what may be a theme for eukaryotic expansion segments in which they function as binding sites for accessory proteins. ES7L of the 28S rRNA interacts with SBP2, a protein involved in selenoprotein synthesis (Kossinova et al., 2014) and with aminoacyl-tRNA synthetases (Krauer et al., 2021), although the consequence of these interactions is unclear. ES7 has also been shown to contain sequences that assemble into G-quadruplexes (G4s) (Mestre-Fos et al., 2019). G4s are non-canonical nucleic acid structures that form following Hoogsteen base-pairing of four guanosines into a G-quartet (Fay et al., 2017). If the sequence of a nucleic acid permits, several G-quartets may stack to form a G4. This analysis shows that the ES7 G4s can mediate interaction with several proteins; however, the function of these interactions also remains elusive. Another striking example of transcript-specific translation regulation by expansion segments was proposed during development (Leppek et al., 2020). In this model, ES9S was proposed to have a role in directing the translation of Hox gene mRNAs. However, these data have recently been called into question based on misannotation of 5′-UTR start sequences (Akirtava et al., 2022).

Decades of work have revealed the roles of rRNA in protein synthesis; however, our knowledge regarding the functions of ribosomes still largely relies on work conducted in prokaryotic systems. In recent years, there have been giant leaps in the ability to resolve ribosome structures in different states (Ben-Shem et al., 2010; Ben-Shem et al., 2011; Anger et al., 2013; Khatter et al., 2015). The coming years can be expected to reveal important distinctions and similarities between prokaryotic and human ribosomes.

## Genomic organization and synthesis of ribosomal RNA under non-pathogenic conditions

Despite similarities in ribosome function, the assembly of ribosomes is dramatically distinct between humans and prokaryotes. Prokaryotic ribosomes can be assembled *in vitro* using purified components (Traub and Nomura, 1968; Nierhaus and Dohme, 1974) and contain three rRNAs: the 23S and 5S in the large subunit and the 16S in the small subunit. Mammalian ribosomes contain four rRNAs: the 28S, 5.8S, and 5S in the large 60S subunit and the 18S rRNA in the small 40S subunit. Additionally, *in vitro* reconstitution from purified components is not possible for any eukaryotic ribosome. It is also worth noting that while the yeast ribosome also contains four rRNAs, the largest rRNA is referred to as the 25S, not the 28S, rRNA.

**FIGURE 1:**
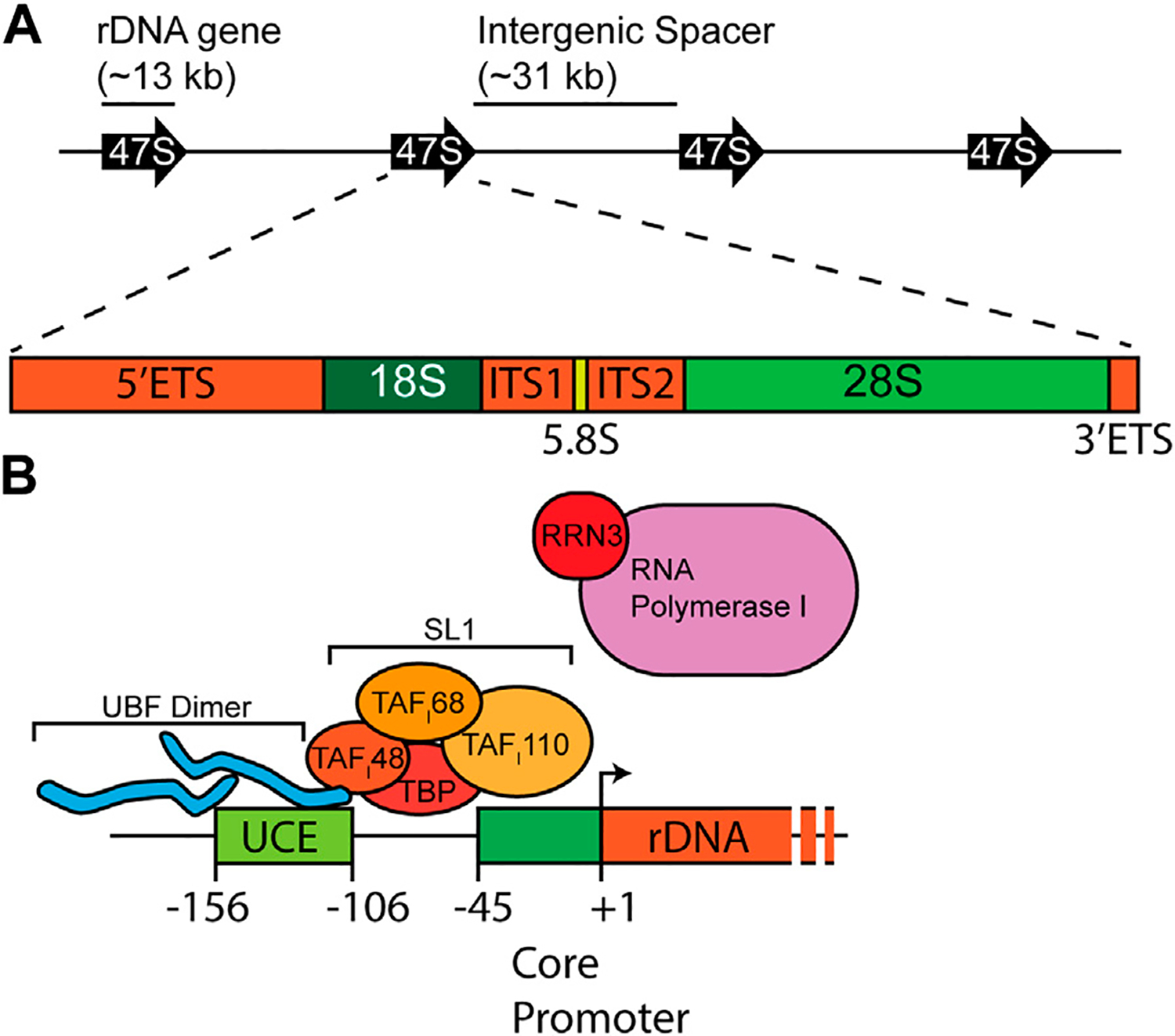
Schema of the rDNA locus and promoter. **(A)** rDNA genes are organized in a head-to-tail configuration. Each gene contains the mature 18S, 5.8S, and 28S rRNA flanked by 5′-and 3′-external transcribed spacers (ETS) and separated by internal transcribed spacers 1 and 2 (ITS1/2). **(B)** Two important sequence elements, the upstream control element (UCE) and the core promoter, drive efficient transcription of rRNA. UBF binds the UCE, while SL1, a multi-subunit complex of TATA-binding protein (TBP) and TAFs, assembles on the core promoter. These proteins work to recruit RNA polymerase I bound by RRN3 for active transcription.

Mammalian ribosome biogenesis predominantly unfolds within the nucleolus, a specialized compartment within the nucleus (Lafontaine et al., 2021). Constituting approximately 80–90% of the total cellular RNA mass, rRNA synthesis primarily relies on RNA polymerase I (Pol I), which synthesizes the tricistronic 47S rRNA precursor, encoding 18S, 5.8S, and 28S rRNAs (Figure 1A). This precursor’s processing is essential for releasing mature rRNAs. The 5S rRNA, produced from monocistronic genes by RNA polymerase III, requires only 5′-end modifications to attain maturity, in stark contrast to the intricate processing of the 47S rRNA.

The synthesis of rRNA emerges as a pivotal bottleneck in the intricate process of ribosome biogenesis (Maden et al., 1969; Emerson, 1971; Liebhaber et al., 1978). Each cell may contain 10^7^ ribosomes that necessitate duplication during cell division to ensure an ample supply for the daughter cells. Failing to achieve this duplication would compromise the cell’s ability to engage in robust protein synthesis. Moreover, this challenge extends beyond duplication as it does not account for the replacement of ribosomes lost due to their natural half-life. Cells adopt a dual-pronged strategy to address the demand for new rRNA synthesis. Actively growing cells exhibit heightened rates of rRNA gene synthesis—a straightforward and effective response. Additionally, cells throughout evolution have expanded their repertoire of rRNA genes. In the case of humans, more than 300 individual copies of the 47S rDNA gene are distributed on the short arms of five acrocentric chromosomes at 13p12, 14p12, 15p12, 21p12, and 22p12 (Henderson et al., 1972; Gaubatz et al., 1976). Traditionally organized in a “head-to-tail” orientation within 43 kb repeated elements, each repeat encompasses a 13 kb rDNA gene and an intergenic spacer (IGS) of approximately 30 kb in length (Figure 1A) (Wellauer and Dawid, 1979). Newer imaging and sequencing approaches have revealed significant variations in rDNA structure and rDNA gene sequences in humans (Caburet et al., 2005; Kim et al., 2018; Nurk et al., 2022). The functional consequences of rRNA variation are still being analyzed.

In humans, the primary cluster for 5S rRNA is located at a specific genomic locus on chromosome 1 at 1q42 (Steffensen et al., 1974; Sorensen et al., 1991). While this cluster boasts over 2000 individual genes, stringent dot-blotting using placenta DNA has indicated that the majority of these may be pseudogenes (Sorensen and Frederiksen, 1991). A more refined assessment revealed that only approximately 400 genes are potentially active. This results in the number of 5S genes roughly aligning with the count of 47S genes. Given that 5S rRNA is equimolarly abundant in ribosomes, this unique genomic organization may serve as a strategic mechanism to balance 5S production with the synthesis of other rRNAs. However, this genomic arrangement is distinctive to higher eukaryotes. In organisms like *Saccharomyces cerevisiae* and *Dictyostelium discoideum*, the 5S rRNA genes are intricately integrated within clusters responsible for transcribing the 18S, 5.8S, and 28S rRNAs (Maizels, 1976; Petes et al., 1978), positioned on the opposite strand of the tricistronic rRNA genes. In contrast, *Schizosaccharomyces pombe* and *Neurospora crassa* exhibit a dispersion of 5S genes throughout their genomes (Selker et al., 1981; Mao et al., 1982).

Despite this expansion, only approximately half of the rDNA repeats appear to be active as the remaining are epigenetically silenced (reviewed in Srivastava et al. (2016)). However, a percentage of active and inactive genes can change in response to stimuli, such as metabolic and environmental signals or stages of the cell cycle. There are three distinct rDNA silencing complexes in humans: the nucleolar remodeling complex (NoRC), the nucleosome remodeling and deacetylation (NuRD) complex, and the energy-dependent nucleolar silencing complex (eNoSC). The NoRC establishes a constitutively silenced rDNA (Strohner et al., 2001). The NuRD complex leaves rDNA promoters in an inactive state but remains accessible for transcription factors, allowing genes to stay “poised” for transcription (Xie et al., 2012). As its name suggests, eNoSC regulates rDNA silencing in response to energy deprivation (Murayama et al., 2008). In mammals, effective silencing is facilitated by the concerted effort of the methylation of histone tails (e.g., H3K4 and H3K79) and by DNA methylation. The repetitive nature of rDNA repeats has historically posed challenges for analyzing the transcriptional status of individual genes, causing a lag in understanding which rDNA genes are selected for silencing and why. However, innovative data analysis approaches and longer read lengths may help bridge these knowledge gaps. Active and silenced genes are interspersed, rather than entire loci being uniformly silenced (Sharp et al., 1984; Dammann et al., 1995; Zillner et al., 2015).

RNA Pol I is specialized for transcribing only the 47S rDNA genes. It is recruited to rDNA promoters which contain two critical regulatory elements: the upstream control element (UCE) and the core promoter (Figure 1B). Initial cell-free transcription experiments from mouse Ehrlich ascites tumor-cell extracts and cloned mouse DNA templates demonstrated that base pairs immediately upstream of the transcription start site, which defined the core promoter, were sufficient to drive transcription (Grummt, 1982). However, these same experiments showed that nucleotides further upstream were important in stimulating the transcription of rRNA when competing with templates containing only core promoters; this upstream region came to be known as the UCE. Later experiments using HeLa extract and human DNA templates validated that human Pol I transcription was also governed by a core promoter and UCE (Learned et al., 1983). Despite the conserved promoter structure across species, there is little promoter sequence homology—a contrast with mature rRNA sequences. In fact, mouse extract is incapable of driving transcription from the human rDNA promoter and vice versa (Grummt et al., 1982).

Identification of these elements initiated the search for the protein factors that acted as *trans* factors. The first of these factors to be identified was termed selectivity factor 1 (SL1) (Learned et al., 1985). SL1 is a multi-subunit complex containing TATA-binding protein (TBP) and multiple transcription activating factors (TAFs) (Comai et al., 1992; Comai et al., 1994; Zomerdijk et al., 1994; Heix et al., 1997; Gorski et al., 2007). However, SL1 has no specificity for the rDNA promoters and no DNA binding activity within the promoter (Bell et al., 1988). DNase I foot-printing experiments showed that SL1 alone would not bind to the rDNA promoters, but partially purified RNA Pol I would associate with the UCE (Learned et al., 1986). The DNA binding activity was further purified from crude RNA Pol I preparations and was termed upstream binding factor 1 (UBF) (Bell et al., 1988). Importantly, UBF and SL1 cooperate to mediate rDNA transcription. The TAF_I_48 subunit of SL1 interacts with UBF (Beckmann et al., 1995), at which point the UBF1 DNA binding targets the complex at rDNA promoters (Bell et al., 1988). Conversely, binding to SL1 stabilizes UBF on DNA (Friedrich et al., 2005).

Another key factor for efficient rRNA transcription initiation is RRN3/TIF-IA. Biochemical fractionation partially purified this protein from mouse cells, based on its ability to stimulate rRNA transcription in growing cells, and it was named TIF-IA (Buttgereit et al., 1985). Later work demonstrated that TIF-IA is regulated by mTOR activity, which is consistent with its loss of activity during reduced growth phases (Mayer et al., 2004). Other work identified the same protein as being able to stimulate rRNA synthesis in response to hormonal stimulation but termed the protein the name TFIC (Mahajan and Thompson, 1990). Yet another group identified the same activity and gave this protein a different name: Factor C* (Brun et al., 1994). It was not until the homologous protein was identified in yeast that this gene was cloned (Milkereit and Tschochner, 1998), and shortly thereafter it was discovered that TIF-IA, TFIC, and Factor C* were the same protein and were the mammalian homolog of yeast Rrn3 (Bodem et al., 2000; Moorefield et al., 2000).

Work from yeast and mammalian systems showed that RRN3/TIF-IA is the limiting factor for rRNA synthesis. Pol I that is unbound to DNA can exist freely or associated with RRN3/TIF-IA. However, only RRN3/TIF-IA-bound Pol I is recruited to DNA to initiate transcription (Peyroche et al., 2000; Blattner et al., 2011), although RRN3/TIF-IA is inactivated and may dissociate following transcription (Brun et al., 1994; Hirschler-Laszkiewicz et al., 2003). Thus, it is likely needed only for DNA recruitment, melting of DNA, and promoter escape (Sadian et al., 2019). Given its key role in regulating rRNA synthesis, RRN3/TIF-IA is a target of many pathways that regulate stress response and growth. In addition to being regulated by mTOR activity, RRN3/TIF-IA is a target of JNK, ERK, RSK, and AMPK (Zhao et al., 2003; Mayer et al., 2005; Hoppe et al., 2009).

## Pathologies arising from defects in rDNA genes or rRNA synthesis

### Treacher Collins syndrome

Treacher Collins syndrome (TCS) is the most common cause of mandibulofacial dysostosis, occurring in 1 in 50,000 live births (Gorlin et al., 1990; Ulhaq et al., 2023). Prior to the identification of the causative gene for TCS, it was noted that this disorder followed an autosomal dominant inheritance pattern but that most cases were sporadic, likely arising from novel private mutations (Jones et al., 1975). Through laborious genetic mapping using short tandem repeat polymorphisms and the generation of yeast artificial chromosomes (YACs) containing the affected genomic region, the major causative gene was identified and named TCOF1 (Dixon et al., 1996). A wide array of seemingly sporadic mutations in TCOF1 have been associated with or are causative of the development of TCS (Wise et al., 1997). Eventually, a mouse model generated heterozygous knockouts of TCOF1, mimicking the disease conditions (Dixon et al., 2006). In this model, there was a defect in neural crest cell migration during development, which led to defects in facial bones, cleft palate, and ear defects consistent with clinical presentations (Jones et al., 2008). The complete analysis of the TCOF1 amino acid sequence led to the hypothesis that it was a possible nucleolar phosphoprotein, based on its similarity to other resident nucleolar proteins (e.g., NOLC1 and NPM) (Wise et al., 1997). This work also noted that TCOF1 displayed particularly low complexity throughout its amino acid sequence, a feature that is now known to aid in the condensation of non-membranous organelles such as the nucleolus (Lafontaine et al., 2021). Experiments formally demonstrated that TCOF1 localized to the nucleolus, and this required sequences in its C-terminus (Winokur and Shiang, 1998; Isaac et al., 2000). A subset of TCS patients do not have mutations in TCOF1. The second and third most common mutations are found in POLR1D and POLR1C, which are subunits of both Pol I and Pol III (Dauwerse et al., 2011). Disease-associated mutations destabilize the function of these enzymes which are critical for the synthesis of rRNAs, emphasizing that TCS arises from a defect in this process (Walker-Kopp et al., 2017).

The first clues about the mechanistic function of TCOF1 emerged as its role in rRNA transcription was revealed. Co-immunoprecipitation and yeast two-hybrid experiments demonstrated that TCOF1 formed a direct complex with UBF (Valdez et al., 2004). Consistent with these data, siRNA-mediated depletion of TCOF1 resulted in a reduced rate of rRNA synthesis. However, data from *Xenopus laevis* also support the hypothesis that TCOF1 may be involved in rRNA methylation, another form of dysfunction found in rRNA synthesis (see below) (Gonzales et al., 2005). However, there is mounting evidence that the rate of rRNA synthesis is linked to the effectiveness of rRNA methylation, so it is unclear whether the effect on rRNA methylation is direct or is a secondary effect from altered rRNA synthesis. However, proteomic analysis of Nop56, a key factor in rRNA methylation, has identified TCOF1 as an interacting factor (Hayano et al., 2003).

### Cockayne syndrome

Cockayne syndrome (CS) is an autosomal recessive disorder that results in diverse clinical presentations. The most common presentations are neonatal failure of growth and neurological dysfunction that worsens with age. However, microcephaly, dwarfism, cardiac defects, increased sensitivity to UV light, and ophthalmologic dysfunction, including retinal degeneration and the increased propensity for cataract formation, are also common (Nance and Berry, 1992). Life expectancy for afflicted individuals is 12 years (Laugel et al., 2008). Fibroblasts from CS patients display reduced proliferation after exposure to UV light based on colony-forming assays (Schmickel et al., 1977). Curiously, these cells appear to have no defect in the rate of DNA repair but have a reduced rate of recovery based on RNA synthesis (Mayne and Lehmann, 1982; Lehmann et al., 1993). In these studies, RNA synthesis was monitored by the uptake of ^3^H-uridine into acid-soluble RNA. Since rRNA accounts for the majority of all cellular RNA synthesis, it can be inferred that these studies monitored the resumption of rRNA synthesis after UV irradiation. Genetic analysis revealed that CS patients fell into at least two distinct complementation groups: CSA or CSB (Tanaka et al., 1981). Clarity regarding the complementation groups and the effect on nucleic acid synthesis came with the isolation of the relevant genes, which were termed CSA (ERCC8) and CSB (ERCC6) (Troelstra et al., 1990; Henning et al., 1995). Both CSA and CSB were determined to associate with transcription factor IIH (TFIIH), which originally implicated a defect in RNA Pol II transcription in CS. However, later data demonstrated that TFIIH was also crucial for Pol I transcription initiation and elongation (Bradsher et al., 2002; Iben et al., 2002; Assfalg et al., 2012). These data, and the dramatic effect on bulk rRNA synthesis, implicated rRNA synthesis as a component of CS pathology.

To date, multiple mechanisms have been proposed for how mutations to CSA and CSB alter rRNA synthesis rates. These mechanisms are not mutually exclusive, and some interplay between proposed mechanisms is possible. TFIIH, which interacts with CSA and CSB, is essential for Pol I transcription initiation in humans and yeast, and it interacts with Pol I and SL1 (Iben et al., 2002; Koch et al., 2014). CSB and TFIIH are also necessary for efficient Pol I elongation (Lebedev et al., 2008; Assfalg et al., 2012). The elongation-promoting activity found in these studies may be related to recent data showing that CSA and CSB can resolve G4 structures (Scheibye-Knudsen et al., 2016; Liano et al., 2021). These are highly stable structures that can block a polymerase if not resolved. Reduction of CSA or CSB leads to the stalling of polymerases at sequences capable of forming G4s. Further analysis shows that CSB can melt these structures, which would allow for more efficient transcription elongation. Surprisingly, chemical stabilization of G4 leads to decreased rRNA synthesis and an increased rate of aging (Scheibye-Knudsen et al., 2016; Liano et al., 2021). These results could tie the rRNA synthesis seen in CS mutations to the pathological aging symptoms in CS patients. CS has also been proposed to function through interaction with nucleolin (NCL) (Okur et al., 2020), which is noteworthy, as NCL has also been implicated in the regulation of G4 structures in rDNA (Drygin et al., 2009; Xu and Hurley, 2022).

Other work has hinted at an alteration of the epigenetic landscape as a mechanism for reduced rRNA transcription. Co-immunoprecipitation studies established that CSB interacts with G9a, a histone methyltransferase that deposits mono- and di-methyl groups on H3K9 (Yuan et al., 2007). The authors propose that these methylation marks aid the recruitment of HP1γ which promotes Pol I elongation. CSB may also interact with the NuRD complex, which establishes a chromatin state that poises individual rDNA genes for transcription (Xie et al., 2012).

Finally, CS is also associated with aberrant ribosome function, although it has not been formally established that this is a consequence of altered rRNA synthesis rates. Nevertheless, cells harboring CS mutations have reduced translation fidelity (Alupei et al., 2018). In these conditions, proteins with misincorporated amino acids have an increased propensity to misfold, which activates the unfolded protein response. Mutations in TFIIH have similar effects on proteostasis and translation fidelity (Khalid et al., 2023). These mutations are associated with trichothiodystrophy (TTD) developmental disorder, which shares common pathologies with CS.

### Alterations to rDNA number in disease

The rDNA repeats are highly recombinogenic (Gaubatz et al., 1976; Kobayashi, 2011). In humans, the average number of rDNA genes is 300, but the absolute number can differ between individuals and within an individual (Johnson and Strehler, 1972; Medvedev, 1972; Johnson et al., 1975; Wang and Lemos, 2017; Malinovskaya et al., 2018). Early work suggested an age-dependent loss of rDNA repeats in brain and heart tissue (Johnson and Strehler, 1972; Medvedev, 1972; Johnson et al., 1975). Follow-up studies have found similar reductions in rDNA number during aging (Zafiropoulos et al., 2005; Ren et al., 2017). This suggested an intriguing hypothesis that an age-related decrease in fitness may be partly due to the loss of proliferative potential by reducing the number of rDNA transcription units. In turn, this would reduce the rate of rRNA synthesis and ultimately ribosome production. In fact, earlier work using *Drosophila melanogaster* genetics had demonstrated that to be true (Ritossa et al., 1966). Correlative work has shown that a low rDNA copy number is associated with mild cognitive impairment and dementia, although these studies could not demonstrate a causative link (Hallgren et al., 2014; Veiko et al., 2022). Expansion and loss of rDNA repeats are associated with human cancers (Wang and Lemos, 2017). In these cases, there is a trend for expansion of the 5S rDNA genes and a decrease in the 47S rDNA genes, leading to a general imbalance. Loss of rDNA repeats is also common in Werner’s syndrome (Caburet et al., 2005), a progeroid syndrome characterized by adult onset rapid aging (Oshima et al., 2017). These are coupled with a wealth of data from *S. cerevisiae* demonstrating an age-dependent loss of rDNA on linear chromosomes and the generation of extrachromosomal rDNA circles (Sinclair and Guarente, 1997). Age in yeast can be measured by replicative lifespan, which is the number of daughter cells a mother cell may produce. Recent data have demonstrated that the loss of rDNA repeats is a major driver of replicative senescence (Hotz et al., 2022).

However, the relevance of this phenotype in humans and its causal effect on aging has been challenged (Gaubatz et al., 1976). A Russian population study demonstrated a narrowing of the range of rDNA copy number with age, but the mean and median number of repeats were unchanged (Malinovskaya et al., 2018).

As the maintenance of rDNA repeats has been implicated in regulating longevity and aging, so has the maintenance of epigenetic rDNA silencing. Overexpression of SIRT1, a component of the eNoSC, has been shown to extend lifespan in *D. melanogaster*, *Caenorhabditis elegans*, and mice (Tissenbaum and Guarente, 2001; Satoh et al., 2013; Whitaker et al., 2013). In contrast, patients with Alzheimer’s disease also show an increase in epigenetic silencing of rDNA repeats, which would result in a reduced transcriptional potential (Pietrzak et al., 2011).

### Alterations to rRNA synthesis in cancer

As loss of rRNA synthesis, either due to epigenetic silencing or rDNA loss, is linked to reduced fitness, increased rRNA synthesis is intrinsically tied to hyperproliferation. The ability to duplicate the cell’s complement of ribosomes is a key factor in its ability to divide, and rRNA synthesis is a rate-limiting step in this process. Some of the earliest histological analyses of cancer cells revealed enlarged nucleoli—the site of rRNA transcription and ribosome biogenesis (Maccarty, 1936). Nearly a century of work has now shown that this is due to an increase in rRNA synthesis.

Stabilization of c-Myc, a protooncogenic transcription factor, is a key feature of carcinogenesis. Transcriptional regulation of RNA Pol II and RNA Pol III genes had been under intense investigation in regulating cell transformation. Data showed that ribosomal protein genes were stimulated by c-Myc (Kim et al., 2000). However, in 2005, three groups independently revealed that c-Myc is also a major driver of rRNA (Arabi et al., 2005; Grandori et al., 2005; Grewal et al., 2005). c-Myc was shown to localize to the nucleus, and its overexpression triggered a dramatic increase in transcription. Conversely, siRNA knockdown of c-Myc reduced rRNA synthesis rates. E-boxes are the DNA element to which c-Myc binds. Analysis of rDNA repeats demonstrates a wide distribution of these sites throughout each repeat, which ChIP experiments demonstrated were bound by c-Myc. Later work showed that c-Myc may reorganize the chromatin to better allow access to SL1 and UBF (Shiue et al., 2009). Recent data also point to UBF mutations as playing a role in acute myeloid leukemia (Stratmann et al., 2021; Umeda et al., 2022; Duployez et al., 2023). Screening of adult and pediatric cases showed internal tandem duplication of exons in UBF. However, the effect of these tandem duplications on rRNA expression is not known.

The increased requirement for *de novo* ribosome production in cancer and metastasis has made inhibition of RNA Pol I an attractive target for anti-cancer therapies. CX-3543 is a small molecule that has been shown to inhibit rRNA synthesis. A proposed mechanism of action is to disrupt nucleolin (NCL) from G-rich sequences in rDNA (Drygin et al., 2009). Under normal conditions, NCL has been proposed to prevent G4 structures from forming, suggesting a mechanism in which CX-3543 displaces NCL from rDNA, leading to an increase in G4 formation and impaired Pol I elongation. This is similar to a proposed cause of Cockayne syndrome. A derivative of CX-3543—CX-5461—was shown to target RNA Pol I more directly (Mars et al., 2020). This drug allows the recruitment of Pol I/RRN3 complexes to promoters but prevents promoter escape and transition to an elongation phase. However, polymerases that have begun elongation are not inhibited, and the validity of this proposed mechanism is a matter of debate. Other work has suggested that the primary mode of action is through the “poisoning” of topoisomerase II (Bruno et al., 2020).

## Mechanism and regulation of rRNA maturation

**FIGURE 2:**
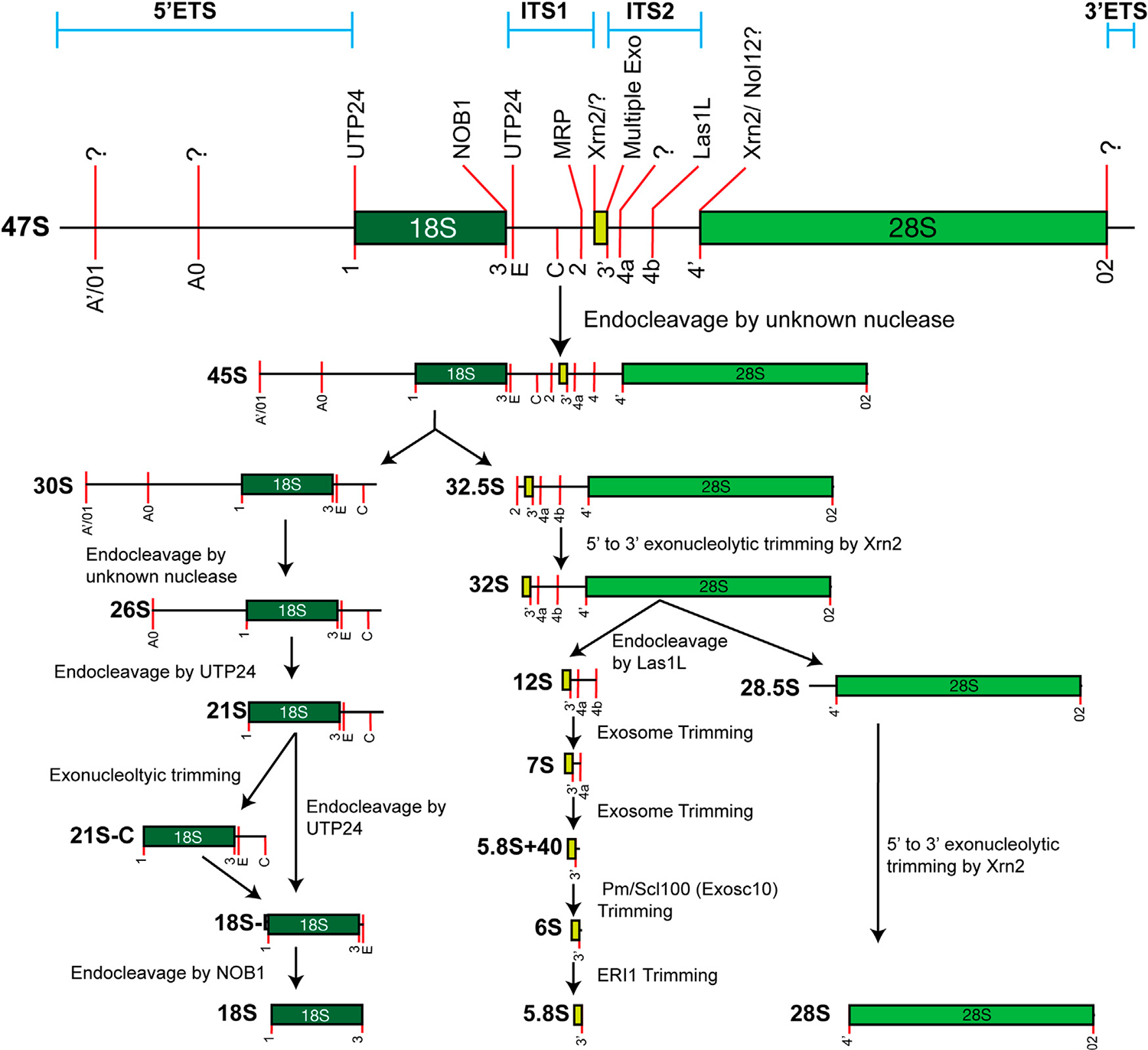
Simplified model of the major 47S rRNA processing pathway. Processing sites and identified processing enzymes are noted on the 47S rRNA, when known. This only schematizes the major pathway. Note that alternative minor maturation pathways exist.

In mammals, the synthesis of four mature rRNAs involves the transcription of two precursor rRNAs. The pre-5S rRNA is transcribed by RNA polymerase III, while the tricistronic 47S pre-rRNA (equivalent to 35S in yeast) is transcribed by RNA polymerase I. Minimal 5′-end processing is required to convert pre-5S rRNA into mature 5S rRNA, whereas the 47S precursor undergoes an intricate maturation process that leads to the release of 18S, 5.8S, and 28S rRNAs (Figure 2). Significant strides in understanding rRNA maturation have been achieved using *S. cerevisiae*, yielding foundational discoveries. However, distinctions between yeast and mammals underscore our incomplete understanding of the maturation process in humans. This review will primarily focus on the maturation of mammalian rRNAs, with a conscientious acknowledgment of pivotal findings derived from yeast studies.

The mature 5S rRNA is the smallest of the four rRNAs. The human 5S is homologous to the prokaryotic 5S rRNA and shares a similar secondary structure. The establishment of *in vitro* transcription systems using isolated human or rat nuclei revealed that the transcription product for the 5S genes was longer than the mature 5S rRNA, suggesting the possibility of a longer precursor (Yamamoto and Seifart, 1978; Hamada et al., 1979). However, these apparent precursors contained extended uridine residues at their 5′-end, so researchers acknowledged the possibility that these arose because of the artificial nature of *in vitro* transcription.

Later work confirmed that this 3′-extended precursor was a natural transcription product. These experiments took advantage of antisera from patients with systemic lupus erythematosus (Rinke and Steitz, 1982). The antisera recognized that the La protein and immunoprecipitation effectively precipitated most Pol III transcripts, including the 5S rRNA. These transcripts were shown to contain similar U-rich 3′-extensions, as had been seen in *in vitro* transcription experiments. Shortly thereafter, mutagenesis experiments established the existence of 5S precursor molecules in yeast (Piper et al., 1983). Similarly, it was discovered that the initial transcripts contained short uridine-rich 3′-extensions. Further work on the mechanism of Pol III termination has shown that these U-rich sequences function as a termination signal, like that seen in prokaryotic transcription (reviewed in Arimbasseri et al. (2013)).

Despite the relatively simplistic processing required to mature the pre-5S to 5S rRNA, the mechanism remains unclear in humans. Work from yeast has shown that the 3′-end of the pre-5S is exonucleolytically processed by Rex1p to generate the mature rRNA (van Hoof et al., 2000). A distantly related protein, Rexo5, appears to be involved in *D. melanogaster* (Gerstberger et al., 2017). However, there are important differences that have yet to be resolved in species-specific processing. In yeast, mutants of Rex1p are viable, whereas mutations to fly Rexo5 are lethal. It is unclear if this is due to added functions of Rexo5 or a reduced stringency in yeast for 3′-extensions. Further complicating this is the unclear role of the mammalian homolog of Rexo5, known as NEF-sp (Silva et al., 2017). In mice, this protein is restricted to gonads, and its loss does not alter viability. The maturation of human 5S rRNA clearly warrants additional research to determine its unique process relative to yeast.

Mammalian tricistronic precursor maturation has been better characterized, although important gaps in knowledge remain. In humans, the most commonly used reference 47S rRNA sequence is 13,304 nucleotides. This sequence is known to contain errors, and increasing evidence supports heterogeneity amongst 47S sequences. The initial transcript contains the 18S, 5.8S, and 28S rRNAs, which are flanked by 5′- and 3′-external transcribed spacers (ETSs). The 18S and 5.8S sequences are separated from each other by internal transcribed spacer 1 (ITS1), while the 5.8S and 28S sequences are separated by ITS2. This general structure is conserved in metazoans, but the spacer regions have greatly expanded in mammals. As with 5S, the presence of precursor molecules from which the 18S, 5.8S, and 28S originated was not a foregone conclusion. The 18S and 28S can be analyzed by radiolabeling RNA and purification by sucrose gradient ultracentrifugation (Philipson, 1961; Scherrer and Darnell, 1962). Brief labeling of RNA identified larger intermediates that are dependent upon active transcription, suggesting that these were precursors of the mature rRNAs (Scherrer and Darnell, 1962; Scherrer et al., 1963; Tiollais et al., 1971).

The first processing events occur nearly simultaneously at the extreme 5′- and 3′-ends to convert the 47S to the 45S pre-rRNA. The 5′-end processing occurs at two closely related sites: A′ and 01 (A’/01). In humans, this site is located approximately 415 nucleotides from the 5′-end of the 47S, although the precise cleavage site is not known. S1 nuclease protection assays and primer extension assays suggest a heterogeneous distribution of cleavage events (Kass et al., 1987). This processing site is not present in yeast, making this system unable to provide further insights into the mechanism of cleavage in humans. Despite these challenges, a pivotal contributor to A’/01 processing is the U3 small nucleolar RNA (snoRNA) (Bachellerie et al., 1983; Crouch et al., 1983; Tague and Gerbi, 1984; Parker and Steitz, 1987; Kass et al., 1990). Notably, this RNA molecule assumes multiple roles in the intricate process of maturing the 18S rRNA. The U3 snoRNA is a member of the Box C/D snoRNA family that primarily guides the 2′-O methylation of rRNA (see below). However, the U3 snoRNA assembles with a host of protein co-factors to then base-pair with the pre-rRNA to direct the processing of the 18S rRNA (Prestayko et al., 1970; Calvet and Pederson, 1981). Evidence suggests that A’/01 processing occurs within minutes (Popov et al., 2013).

Processing at the 3′-end of the 47S is less clear in humans. A processing site known as 02 is found at the 3′-end of the mature 28S. However, it is not known if this is generated by an exo- or endonucleolytic processing event. In yeast, the concerted efforts of an endonuclease—Rnt1p—and exonuclease—Rex1p—are required (Kempers-Veenstra et al., 1986; Kufel et al., 1999). As with 5′-end processing, a snoRNA has been implicated in this processing reaction in vertebrates and mammals. Work in *Xenopus* oocytes demonstrated that the U8 snoRNA was involved in this process (Peculis and Steitz, 1993), and later work has confirmed that this is conserved in mammals (Srivastava et al., 2010; Langhendries et al., 2016).

Although minor processing pathways exist in metazoans, the next major processing event is endo-nucleolytic cleavage in ITS1, which separates the small and large subunit rRNAs. This cleavage event at Site 2 is mediated by the MRP complex, a ribonucleoprotein that contains the RMRP catalytic RNA (see below for additional details).

Maturation of the 18S requires removal of the 5′-ETS and the 5′-portion of ITS1 after cleavage. Like cleavage at A’/01, the discrete processing requirements for the removal of 5′-ETS still require clarification. However, they are dependent upon the U3 snoRNP, which aids in the assembly of the small-subunit (SSU) processome. There are two identified processing sites remaining in the 5′-ETS after A’/01 cleavage—A0 and 1—which may be functionally related to the A_0_ and A_1_ sites in yeast. This supposition is strengthened by the fact that Site 1 in humans and A_1_ in yeast is cleaved by homologous protein Utp24, a PIN (PilT N terminus) domain-containing endonuclease. Utp24 and other U3 proteins (Utp) are associated with the U3 snoRNP (Bleichert et al., 2006). Genetic evidence, crosslinking, and *in vitro* cleavage experiments in yeast and humans have established that Utp24 performs this processing (Bleichert et al., 2006; Wells et al., 2016). Some evidence has suggested that another UTP, Utp23, may be involved in both A0 and A_0_ cleavage in humans and yeast, respectively. However, in yeast, the catalytic nucleotides are not required for efficient cleavage, suggesting that Utp23 serves a structural role (Bleichert et al., 2006; Hoareau-Aveilla et al., 2012). In humans, the catalytic PIN domain does appear to have a role in promoting cleavage, though it is yet to be validated if Utp23 is directly responsible (Wells et al., 2017).

Utp24 also has a role in generating the mature 3′-end of the 18S rRNA by cleaving at Site E to generate the 18S-E precursor (Bleichert et al., 2006; Wells et al., 2016). However, the ITS1 fragment may be exonucleolytically trimmed by the exosome before Utp23 cleavage to generate the 21S-C precursor (Carron et al., 2011). It is not clear if this is an obligatory step in processing, although the 21S-C precursor is stabilized in patients with Diamond–Blackfan anemia, a ribosomopathy that arises due to mutations in ribosomal proteins. 18S-E contains approximately 78 extra nucleotides at its 3′-end relative to the mature rRNA. Unlike other steps in this process, the final maturation is completed in the cytoplasm by Nob1, another PIN-domain-containing protein (Udem and Warner, 1973; Rouquette et al., 2005). Various proteins, including PNO1, a binding partner of Nob1, ensure that pre-40S ribosomes do not prematurely engage with the translation machinery before final maturation (Tone and Toh-e, 2002; Lebaron et al., 2012). Phosphorylation of PNO1 by RIOK1 triggers its displacement from pre-40S ribosome and allows for final 18S maturation by Nob1 (Ameismeier et al., 2020).

Maturation of the large subunit rRNAs requires separation of the 28S from the 5.8S rRNA via cleavage in ITS2 and final 5′- and 3′-end processing of each mature rRNA. The endonuclease Las1L cleaves within ITS2 to separate the two immature rRNAs, requiring the polynucleotide kinase activity of Grc3/Nol9 (Gasse et al., 2015; Fromm et al., 2017; Pillon et al., 2017; Pillon et al., 2018; Pillon et al., 2019). The majority of these analyses have been conducted in yeast, but it is presumed that their function is conserved in humans. Yeast Grc3 recruits Las1 to the site of cleavage and “programs” it by promoting its proper conformation. In humans, RNAi-mediated depletion of these proteins blocks ITS2 cleavage (Castle et al., 2010; Heindl and Martinez, 2010). Las1L and Nol9 also assemble into a complex whose components are necessary for ITS2 processing (Castle et al., 2012).

The 5′-end of the 5.8S rRNA is likely matured primarily through the action of exo-nucleolytic trimming. This generates two distinct 5′-ends, resulting in the longer 5.8S_L_ or the 5.8S_S_, which differ by seven nucleotides (Henry et al., 1994). While most mechanistic details were discovered in yeast, the basic processes appear to be conserved in humans. The 5′- to 3′-exonuclease Rat1 is responsible for 5.8S 5′-end maturation in yeast (Amberg et al., 1992; Xue et al., 2000). Structurally, human Xrn2 is homologous to yeast Rat1 (Shobuike et al., 1995), and its depletion impairs 5′-end maturation of the 5.8S rRNA (Wang and Pestov, 2011). These same proteins are responsible for 5′-end maturation of the 28S rRNA after ITS2 cleavage (Geerlings et al., 2000; Wang and Pestov, 2011).

Finally, the maturation of the 3′-end of the 5.8S rRNA is a concerted effort of several exonucleases that generate discrete intermediates. The major player in this process is a multi-subunit complex known as the exosome (Mitchell et al., 1997). Human exosome was first characterized using autoantisera from patients with scleroderma, while the yeast exosome was identified genetically (Reimer et al., 1986; Allmang et al., 1999; Brouwer et al., 2001). The Exo9 complex is the structural unit of the exosome that contains nine polypeptides in a barrel-like structure. Each polypeptide has features of an exonuclease, but catalytic activity is granted by adding one or two additional proteins—Exosc10 (Pm/Scl100) and Dis3—to form the Exo10 or Exo11 complex. The exosome trims the largest 5.8S precursor, known as the 12S, to generate the 7S precursor. This processing may also involve a second exonuclease called interferon-stimulated 20-kDa exonuclease-like 2 (ISG20L2) (Coute et al., 2008). The exosome then completes the next processing reaction to generate the 5.8S + 40 precursor. As the name implies, this precursor contains an additional 40 nucleotides at the 3′-end of the 5.8S rRNA. This length is dictated by the distance an RNA must traverse through the Exo9 barrel before reaching the catalytic subunits (Schuller et al., 2018). At this point, Exosc10 can function independently of the exosome complex to trim the 5.8S + 40 to generate the final precursor, known as the 6S. As with the final 3′-end-maturation of the 18S, the final processing event occurs in the cytoplasm. The multifunctional exonuclease Eri1 (3′-hExo) removes the final nucleotides in the cytoplasm (see below for additional details).

### Cartilage–hair hypoplasia

Cartilage–hair hypoplasia (CHH) is a type of dwarfism first described by McKusick in Old Order Amish populations (McKusick et al., 1965). CHH is a pleiotropic disease causing a variation of clinical presentations that include immune dysfunction and increased cancer risk (Ridanpaa et al., 2001). The etiology of CHH was unknown until the late 1990s, when a candidate genomic region was mapped to chromosome 9 using linkage analysis on genomic data from affected Finnish families (Sulisalo et al., 1993). This region was further narrowed down to Chr9p13 using both linkage and physical mapping techniques (Sulisalo et al., 1994; Vakkilainen et al., 1999). The causative gene remained elusive until 2001, when an insertion in *RMRP* was discovered in a patient suffering from CHH. The *RMRP* gene encodes an untranslated RNA that acts as the RNA component of the ribonuclease RNase MRP (Ridanpaa et al., 2001).

Ribonuclease mitochondrial RNA processing (RNase MRP) was first identified in 1987 as a ribonuclease responsible for the cleavage of mitochondrial RNA transcribed during replication in mice and humans. It was subsequently discovered that RNase MRP has an RNA component encoded in the nucleus that is required for activity (Chang and Clayton, 1987a; Chang and Clayton, 1987b). Despite having mitochondrial processing functions, RNase MRP was predominantly localized to the nucleolus, suggesting an alternative role outside of mitochondria (Yuan et al., 1989; Topper and Clayton, 1990; Karwan et al., 1991; Kiss et al., 1992; Schmitt and Clayton, 1992). Due to its known processing roles, it warranted further investigation as an rRNA processing factor. *RMRP* is also referred to as 7–2 RNA and *NME1* (Kiss et al., 1992; Schmitt and Clayton, 1992). Interestingly, RNase MRP has a conserved secondary structure similar to that of RNase P, which plays a role in tRNA processing. Taken together, its nucleolar localization and RNA cleavage activity suggested that RNase MRP was acting as an rRNA processing enzyme (López et al., 2009).

This supposition was strengthened by extensive studies in *S. cerevisiae*. Two temperature-sensitive (*ts*) mutants, coined *rrp2*-1 and *rrp2-2*, were found to display impaired rRNA processing (Shuai and Warner, 1991; Lindahl et al., 1992). Northern blotting studies revealed an abnormal processing of yeast 35S rRNA. Further characterization of *ts*-mutants revealed misprocessing of the 5.8S rRNA, with the major effect being a non-canonical product that was extended by 149 nucleotides. They also determined there was an alteration to the stoichiometry between 5.8S_L_ and 5.8S_S_ forms. (Lindahl et al., 1992). Two years later, the *NME1* gene was shown to complement the *rrp2-2* mutants. By deleting portions of the *NME1* gene at various lengths (14, 22, and 65 nucleotides), it was found that longer deletions prevent the ability of *NME1* to complement *rrp2-2* mutations. These data provided evidence that *rrp2-2* mutants were actually *NME1* mutants (Chu et al., 1994). Studies in *S*. *cerevisiae* revealed that *NME1*, the gene homologous to human *RMRP*, was required for viability (Schmitt and Clayton, 1992). Schmitt and Clayton (1993) later confirmed that RNase MRP/NME1 in the nucleus is required for appropriate processing of yeast 5.8S rRNA. The complete processing activity of RNase MRP in *S. cerevisiae* was proposed shortly after these studies. *In vitro* experiments revealed that yeast RNase MRP-mediated processing of pre-rRNA is achieved through cleavage at the A_3_ site, which is located in ITS1 (Henry et al., 1994; Lygerou et al., 1996). It was also shown that mutations in POP1, a protein component of the RNase MRP RNP, cause defects in pre-rRNA processing similar to RNase MRP mutants, as well inhibiting A_3_ cleavage (Lygerou et al., 1994).

It took significantly longer to clarify the role of *RMRP* in humans and the connection to CHH, but this has now been validated to direct the analogous cleavage event at site 2 in ITS1 of the human pre-rRNA. Earlier studies initially shed doubt on whether RMRP served a similar role as yeast NME1. RNAi-depletion of POP1, RPP38, or RPP40 protein cofactors of RNase MRP and RNase P did not show alterations in rRNA processing (Sloan et al., 2013). However, the Cech group later concluded that siRNA or antisense oligonucleotides were not sufficient to disrupt RMRP and used CRISPR-Cas9 to edit RMRP (Goldfarb and Cech, 2017). In agreement with yeast studies, complete ablation of *RMRP* is lethal. An increase in rRNA precursors, specifically on the 5′-end of the 5.8S, was observed in HeLa clones that did accumulate *RMRP* mutations.

In CHH, two main classes of mutation have been described; the first consists of either insertions or duplications that occur between the promoter and the transcription initiation site. It is thought that these mutations prevent the effective transcription of *RMRP*. The second category consists of single-nucleotide substitutions that are generally found in conserved regions of *RMRP*. These mutations, primarily A70G and G262T, are thought to have functional consequences (Ridanpaa et al., 2001). The authors also found that all patients with mutations in *RMRP* express RNase MRP to some degree, but patients who are heterozygous for 5′-end insertions have one allele silenced, resulting in lower expression of RNase MRP RNA; this indicates that the 5′-insertion mutations do, in fact, suppress transcription. Co-immunoprecipitation studies show that proteinaceous subunits still interact with mutant RMRP (Ridanpaa et al., 2001). In contrast with yeast *rrp2-2* mutants, CHH patients do not present with the 149 nt extended 5.8S. Stabilization of such an rRNA may be inviable outside of the context of *ts*-mutants.

Recent studies have described A70G mutations resulting in a decreased abundance of cytosolic ribosomes, as well as a reduction in rRNA on a per-cell basis (Robertson et al., 2022). While previous studies found abnormalities in pre-rRNA processing, effects on ribosomal biogenesis of abundance were not previously described. Robertson et al. (2022) has cemented CHH as a *bone* fi*de* ribosomopathy. A previously uncharacterized mutation in the gene *NEPRO* was recently described as causing a CHH-like phenotype. While *NEPRO* is thought to interact with *RMRP* components, these studies are still in their infancy and warrant further investigation (Narayanan et al., 2019; Remmelzwaal et al., 2023).

CHH has been primarily investigated on the level of pre-rRNA processing, although only recently has it been studied at the level of ribosome abundance and biogenesis. It would be interesting to explore with new biochemical tools whether these ribosomes are translation competent and, if so, what their translation capacity and efficiency is. While the pre-rRNA processing role in CHH has been well established, understanding downstream effects on translation would be a significant continuation of research.

### North American Indian childhood cirrhosis

North American Indian childhood cirrhosis (NAIC) is a familial disease displaying an autosomal recessive inheritance that specifically affects the Oji-Cree people of Northwestern Quebec (Betard et al., 2000). The disease is characterized by neonatal intrahepatic cholestasis, jaundice, and progressive liver damage (Weber et al., 1981).

Using genome-wide scanning techniques and linkage analysis on samples from affected and unaffected individuals, the locus responsible for this condition was mapped to chromosome 16q22 (Betard et al., 2000). Just 2 years after identifying the locus, the gene implicated in NAIC was found, which encoded an uncharacterized protein referred to as *FLJ14728*—now called *Cirhin/CIRH1A*. This protein is homologous to yeast UTP4, which is a component of the SSU processome. Homozygous individuals who had the R565W mutation (c.1741C>T), located in the C-terminal end of the protein, were identified. Conservation of the *Cirhin* gene has also been shown across vertebrates and specifically the presence of a dibasic residue in the 565 position (Chagnon et al., 2002).

As *Cirhin* was previously uncharacterized, its normal function was largely unknown. It was found that both wildtype and R565W mutant *Cirhin* localize to the nucleolus. Interestingly, the nucleolus is not generally implicated in diseases of intrahepatic cholestasis, suggesting a potential function for *Cirhin* in the nucleolus (Yu et al., 2005). In *S*. *cerevisiae*, U3 snoRNA and associated proteins (t-Utps) are required for processing of 18S pre-rRNA. In the absence of t-Utps, production of mature 18S rRNA is abrogated (Dragon et al., 2002). While the function of t-Utps has been well established in pre- 18S rRNA processing, it has also been characterized as linking its ribosomal RNA processing function with the transcription of rDNA (Gallagher et al., 2004). Through TBLASTN searches, potential human orthologs of several yeast t-Utps were identified: *hUTP4*, *hUTP5*, *hUTP10*, *hUTP15*, and *hUTP17*, with *Cirhin* being *hUTP4*. These UTPs comprise the SSU processome (Prieto and McStay, 2007). Given that CIRHIN/Utp4 are part of the SSU processome, this may indicate potential processing defects of rRNA in the small ribosomal subunit.

To further elucidate the functions of CIRHIN and its role in the pathogenesis of NAIC, yeast two-hybrid (Y2H) screens and mass spectrometry (MS) were performed to identify potential binding partners for CIRHIN. NOL11, another member of the human t-Utp complex, was found to be a binding partner with CIRHIN. To further parse this, co-immunoprecipitation revealed that NOL11 is part of the SSU processome based on its co-immunoprecipitation with FBL. Interestingly, NAIC mutations (R565W) in CIRHIN ablate interaction with NOL11 (Freed et al., 2012). In addition to association with CIRHIN, siRNA knockdown of NOL11 indicates that it is required to generate mature 18S rRNA (Freed et al., 2012). While it has been shown that hUTP4 with the R565W mutation can localize to the nucleolus normally (Yu et al., 2005), it is reported that CIRHIN interaction with NOL11 may be required for appropriate localization, as C-terminal truncations of CIRHIN prevent nucleolar localization (Freed et al., 2012). While the R565W mutation in hUtp4 appears to interfere with NOL11 interaction, it is curious that nucleolar localization still occurs despite the potential weakening of this interaction. While biochemical data are beginning to reveal the function of this protein, the precise effect on rRNA production and ribosome activity in NAIC awaits clarification.

### ERI1: an emerging disease target

ERI1 (3′-hExo) is a multifunctional member of the DEDDh family of exoribonucleases that possesses 3′- to 5′-exonuclease activity. Amongst other activities, it conducts the final processing reaction to generate the mature 3′-end of the 5.8S rRNA. Originally identified via genetic screening of *C. elegans* mutants with a sensitivity to dsRNA, the ERI1 gene was identified as a potentially important regulator of siRNA. It was found that mutations in eri-1 result in an accumulation of siRNAs, potentially pointing toward regulatory role for ERI1 in siRNA levels. In fact, ERI1 acts as a 3′- to 5′-nuclease and can degrade siRNAs at the 3′-end (Kennedy et al., 2004). This nuclease function against siRNAs is found to be conserved in humans and the fission yeast *S. pombe* (Kennedy et al., 2004; Iida et al., 2006). In addition to negatively regulating siRNAs, ERI1 has also been shown to degrade the 3′-end of histone mRNAs, as well as 3′-end processing of the 5.8S rRNA (Gabel and Ruvkun, 2008). Despite its low sequence homology, structural studies using crystallography and characterization of its nuclease activity confirm its identity as a DEDDh nuclease (Dominski et al., 2003; Yang et al., 2006).

Given the characterized function of ERI1 as a 3′-end processing enzyme of the 5.8S rRNA in *C. elegans*, (Gabel and Ruvkun, 2008), it appears that this function is conserved to mouse ERI1, as ERI1-deficient mice exhibit a longer 5.8S rRNA and can be rescued by reintroducing wild-type Eri1. Biochemical *in vitro* processing studies indicate that recombinant ERI1 can catalyze the final 3′-end processing required for the appropriate formation of the 3′-end of 5.8S. ERI1 performs its 3′-end processing activities in a duplex formed by a 5.8S and 28S rRNA molecule, and subsequently chewing back unpaired nucleotides at the 3′-end of the 5.8S rRNA (Ansel et al., 2008). In addition to carrying out processing activities of the 5.8S, ERI1 also appears to associate with ribosomal proteins and co-sediments with the 40S, 60S, and 80S peaks, but is depleted from actively translating polysomes. While the rRNA processing function has been characterized, the significance of sustained association with the ribosome after processing is unclear (Ansel et al., 2008).

Aside from rRNA processing functions, ERI1 is also necessary for the efficient decay of histone mRNAs at the end of the S-phase (Dominski et al., 2003). Histone mRNAs are the only cellular mRNAs that are not polyadenylated, instead ending in a conserved stem-loop. At the end of the S-phase, when bulk histone production is no longer necessary, they are rapidly degraded. ERI1 binds to the histone stem-loop and participates in the early stages of degradation.

In recent years, an emerging set of data implicates ERI1 in several disease types. Skeletal and cardiac malformations and intellectual disability were reported in an individual with a homozygous deletion of *ERI1*, *MFHAS1*, and *MIR4660* (miR-4660). While *ERI1* deletion likely plays a role in this pathology, it cannot solely be attributed to it (Choucair et al., 2017). Another patient was also identified recently as having a homozygous nonsense mutation (K188Stop) (Hoxha and Aliu, 2023). Similarly, this patient presented with limb abnormalities and intellectual disabilities, strengthening the case that ERI1 loss was the disease-causing mutation in the previous case. Finally, a recent study contrasts clinical manifestations in patients with either missense variants or bi-allelic null mutations in *ERI1* (Guo et al., 2023). Patients with missense variants tend to have major skeletal defects known as severe spondyloepimetaphyseal dysplasia (SEMD), while those with biallelic null mutations suffer from much milder skeletal malformations and intellectual disabilities. Patients with *ERI1* mutations exhibit defective processing of the 3′-end of 5.8S rRNA, as well as accumulating replication-dependent histone mRNAs (Guo et al., 2023). While these recent cases strongly implicate ERI1 in disease, the cause of these diseases is not known. Since ERI1 has many RNA targets, it is unclear whether these conditions arise from the misprocessing of rRNA, a defect in histone mRNA decay, or the stabilization of other uncharacterized RNAs. Future work is needed to determine the mechanistic causes of these disease states.

## Modification of ribosomal RNA under non-pathogenic conditions

**FIGURE 3:**
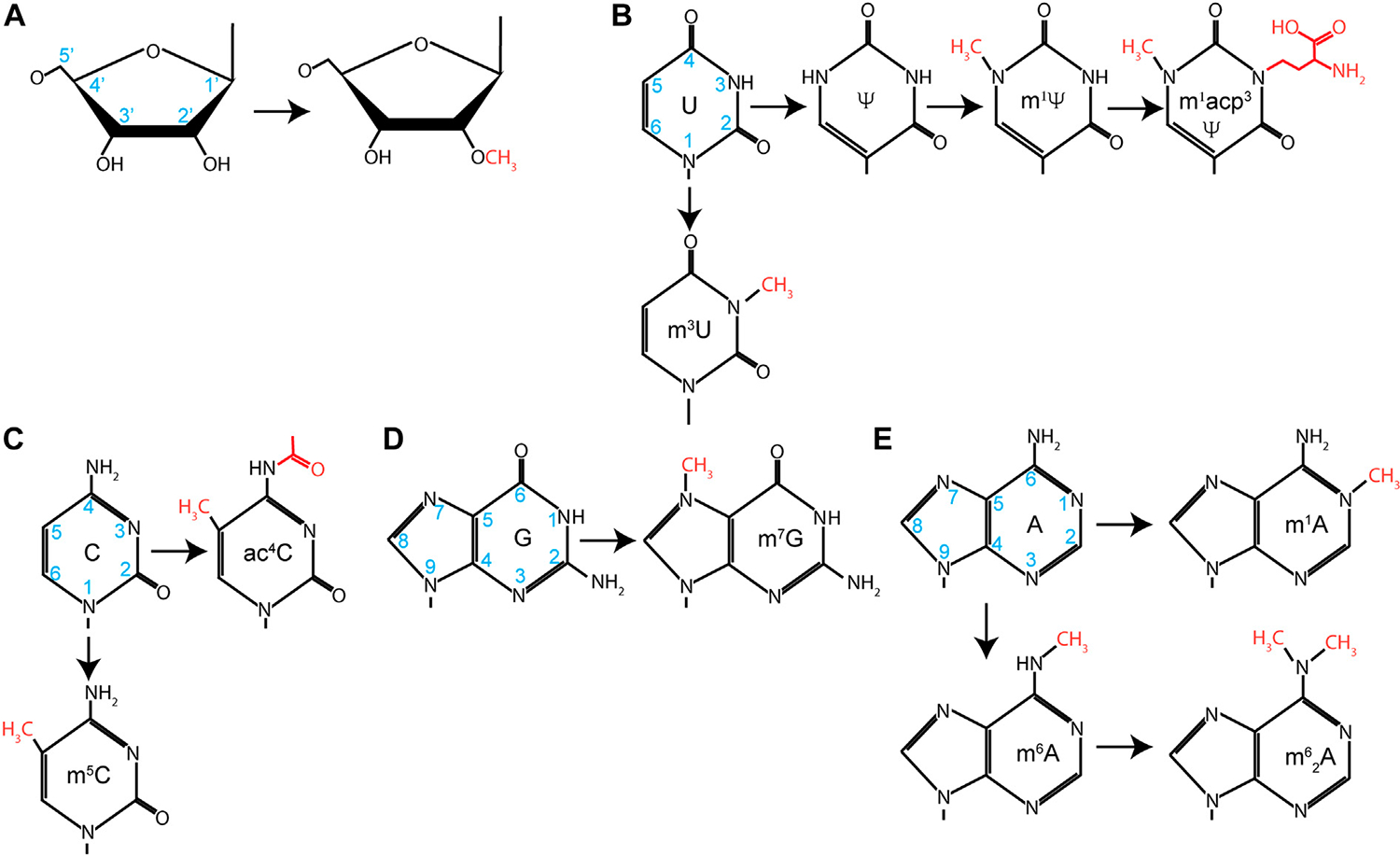
Nucleotide modifications found in rRNA. Chemical modification found on **(A)** ribose, **(B)** uridine, **(C)** cytosine, **(D)** guanosine, or **(E)** adenosine. Carbon number is noted on unmodified nucleotides in blue, and additional groups added during modification are identified in red.

Production of mature rRNAs requires both efficient processing and elaborate chemical modifications. rRNAs are second only to tRNAs in the number and diversity of chemical modifications deposited on the RNA (Figure 3). Most of these modifications are 2′-O methylations (2′-Ome) and pseudouridylations (Ψ), although other base modifications, such as methylations and acetylations, are also present. While these modifications can be found throughout an rRNA, they are clustered around active sites on the ribosome, such as the decoding center and the peptidyl transferase center. The majority of the functional experimental data are derived from yeast and prokaryotic work, but data from various human disease states strongly suggest that these results also hold true for humans. It has been shown for 2′-Ome and Ψ modifications that loss of one or two modifications often has little impact on ribosome structure or function. However, defects begin to emerge as increasing modifications are lost. A seminal study from yeast analyzed modifications found in helix 69 of the 25S rRNA (Liang et al., 2007); helix 69 interacts with helix 44 of the 18S rRNA to form an inter-subunit bridge between the 40S and 60S ribosome and is well conserved from prokaryotes to humans (Yusupov et al., 2001). This helix contains several modifications over an 11-nucleotide stretch (AmCΨAΨGACCΨCΨ). Preventing the deposition of the modifications in this region has a severe effect on protein synthesis and growth (Liang et al., 2007). Yeast ribosomes lacking these modifications have a loss of translational fidelity, a decreased rate of protein synthesis, an increased rate of turnover, and a decreased rate of rRNA processing. Data from prokaryotes demonstrate that the Ψ modifications are important for the proper structure of helix 69 (Jiang et al., 2014). Other work analyzed 18S rRNA modifications present within the decoding center and found similar perturbations to ribosome function and maturation (Liang et al., 2009). More recent data analyzing only 2′-Ome and Ψ modifications highlight the global role of these modifications in accurate translation and in mediating proper ribosome dynamics (Khoshnevis et al., 2022; Zhao et al., 2023).

Functionally, we are still gaining new knowledge about how these modifications regulate translation, although their chemical role is broadly clear. The 2′-OH groups of the rRNA are hydrophilic, which encourages preferential orientation away from the more hydrophobic core of the ribosome. Methylation of this group increases the hydrophobicity of the nucleotides, allowing them to be buried within the ribosome, and stabilizes RNA helices. Isomerization of uridine by 180° rotation around C_6_ converts U to Ψ. This generates a carbon–carbon glycosidic bond and frees the N_1_ to take part in additional hydrogen bonding interactions, further stabilizing the ribosome structure.

rRNA modifications are a significant source of ribosome heterogeneity. This is a hypothesis that not all ribosomes are identical and that the cellular pool of ribosomes is more heterogeneous than previously thought. In recent years, this hypothesis has gained widespread acceptance (Xue and Barna, 2012; Genuth and Barna, 2018). Further speculation has proposed that some ribosomes may have specialized activities in cells by actively translating distinct subsets of mRNAs. The existence of such specialized ribosomes is still a matter of debate. While some studies strongly suggest the existence of specialized ribosomes, the field awaits definitive proof (Ferretti and Karbstein, 2019). Much of these data have focused on alterations of the protein complement of ribosomes, either through differences in core r-proteins or through association with different ribosome-associated factors, although alterations in rRNA modifications have now been seen in various organisms. Ribo-meth seq revealed that several sites of 2′-O methylation are sub-stoichiometric and that the stoichiometry of modification changes depending upon cell type, leading to the proposal that it may provide the basis for differential regulation through methylation (Birkedal et al., 2015; Krogh et al., 2016). This has been given further credence by a quantitative mass-spectrometry approach showing that other modifications, such as Ψ and base methylations (e.g., m^1^A), are also sub-stoichiometric at some positions (Taoka et al., 2018). Furthermore, there are developmentally regulated changes in rRNA methylation in mice (Hebras et al., 2020). Recent work has even shown that these developmental changes are important for proper cellular differentiation in mouse embryos (Hafner et al., 2023). Loss of U_3904_ 2′-O methylation on the 28S rRNA drives mouse embryonic stem cells toward neuroectodermal cell fate.

In metazoans, 2′-Ome and Ψ modification are catalyzed by small nucleolar ribonucleoproteins (snoRNPs) (Ojha et al., 2020). SnoRNPs fall into two classes based on their structure, associated proteins, and which chemical modifications they catalyze: Box C/D snoRNPs and Box H/ACA snoRNPs. Box C/D snoRNPs catalyze 2′-Ome, while Box H/ACA catalyzes Ψ deposition. SnoRNPs are assemblages of catalytic protein subunits and snoRNA subunits that guide chemical modification through Watson–Crick base-pairing. Fibrillarin (FBL) assembles onto Box C/D snoRNAs along with SNU13, NOP58, and NOP56. FBL is a methyltransferase that uses *S*-adenosylmethionine as a donor to deposit methyl groups. Alternatively, dyskerin (DKC), a pseudouridine synthetase, assembles on Box H/ACA snoRNAs with NHP2, NOP10, and GAR1 to form the active Box H/ACA snoRNP. The assembly of these snoRNPs and their functional regulation have undergone intense study, initially in archaea and yeast systems and eventually in mammals (Ojha et al., 2020). However, new discoveries are still being made. *N*^*4*^-acetylation of C_1773_ (ac^4^C_1773_) is required for ribosome biogenesis in yeast and is deposited by KRE33 (Ito et al., 2014a). In humans, NAT10 was identified as the acetyltransferase required for this event at C_1337_ and C_1842_ of the 18S (Ito et al., 2014b; Sharma et al., 2015). Surprisingly, the U13 snoRNP was shown to guide this modification. The U13 snoRNA is a Box C/D snoRNA, but it has not yet been shown to guide 2′-O methylation of any other nucleotide. Thus, it is likely that this snoRNA has evolved to direct the acetylation rather than the methylation of rRNA.

This highlights the fact that, while the majority of modifications are 2′-Ome and Ψ, others, including ac^4^C, are present. Simple base modifications such as 7-methylguanosine (m^7^G) and 1-methyladenosine (m^1^A) are typically carried out by standalone methyltransferases. More elaborate modifications, such as 1-methyl-3-(3-amino-3-caboxypropyl) pseudouridine (m^1^acp^3^Ψ), take the concerted efforts of several modification enzymes. In the case of m^1^acp^3^Ψ, uridine is first isomerized to Ψ, at which point a methyl group is added to the *N*1 position by EMG1 (Wurm et al., 2010; Meyer et al., 2011). Finally, a 3-amino-3-carboxypropyl moiety is added by TSR3 (Meyer et al., 2016). Recent evidence points to this modification as being important in the development of cancer in addition to previous evidence highlighting this modification in Bowen–Conradi syndrome (see below).

### Dyskeratosis congenita

X-linked dyskeratosis congenita (XDC), also known as Zinsser–Cole–Engman syndrome, is a rare multisystem disease presenting clinical features such as reticulate skin hyperpigmentation, nail dystrophy, bone marrow failure, oral leukoplakia, and an increased risk of cancer (Savage et al., 1993). The original name for the syndrome suggests an X-linked pattern of disease inheritance, although a haplo-insufficient autosomal dominant and autosomal recessive mode of transmittance has since been demonstrated. A second disorder, Hoyeraal–Hreidarsson (HH) syndrome, is considered a severe form of XDC that is associated with cerebellar hypoplasia, intrauterine growth retardation (IUGR), and immunodeficiency (Hoyeraal et al., 1970; Aalfs et al., 1995).

Earlier genetic analysis linked the causative gene for XDC to Xp28 (Connor et al., 1986). Later analysis determined that the vast majority of XDC and HH patients have mutations in the *DKC* gene (Heiss et al., 1998; Knight et al., 1999). DKC is the catalytic subunit of Box H/ACA snoRNP that catalyzes the isomerization of uridine to pseudouridine in rRNA and is the human homolog of yeast *S. cerevisiae* Cbf5 (Meier and Blobel, 1994; Cadwell et al., 1997). However, mutations in other Box H/ACA-associated factors—NOP10 and NHP2—have been shown to cause disease with similar clinical presentations (Walne et al., 2007; Vulliamy et al., 2008; Benyelles et al., 2020).

Despite the clear role of DKC in ribosome biogenesis, the cause of symptoms in XDC patients has been a matter of debate due to the assembly of DKC in the non-canonical snoRNP telomerase RNA component (TERC). Importantly, TERC is necessary for the maintenance of telomeres (Heiss et al., 1998); patients with XDC also have defects in telomere maintenance (Mitchell et al., 1999). However, XDC patients also have a marked decrease in rRNA pseudouridylation and defects in translation. Model system data have reported conflicting data, with mouse models recapitulating disease while primarily altering ribosome dynamics with no immediate loss of telomere length (Ruggero et al., 2003); however, work on iPSCs demonstrates the opposite (Gu et al., 2015). Furthermore, mutations in the RNA component of telomerase (hTR) cause disease similar to XDC (Vulliamy et al., 2001), which would implicate telomerase maintenance as the major culprit in XDC etiology. Mutations in nucleophosmin, another ribosome biogenesis factor, perturb 2′-Ome rather than Ψ and cause XDC (Nachmani et al., 2019). These data support the model in which alterations to rRNA modifications are the major driver of disease. Recent data have also implicated mutation of DKC and NOP10 in nephrotic syndrome (Balogh et al., 2020). Like XDC, these mutations result in a defect in telomere maintenance and in ribosome activity. However, the clinical presentations are distinct, further highlighting the pleiotropic role of DKC mutations. Thus, it is likely that the clinical symptoms arise from an additive effect of telomere and translational dysfunction.

The global reduction of Ψ in rRNA, as seen in DKC, is intrinsically linked with translational dysfunction as are site-specific losses of Ψ (Liang et al., 2007; Penzo et al., 2015).

A mouse model of XDC demonstrates diverse defects in ribosome function, demonstrating a loss of Ψ deposition and a reduced rate of rRNA maturation (Ruggero et al., 2003). This same model system reveals a defect in the translation of p27, BCL-xL, and XIAP mRNAs and a concomitant decrease in p27, BCL-xL, and XIAP protein levels in DKC-mutant cells (Yoon et al., 2006; Bellodi et al., 2010a). Further work also implicated alterations in p53 mRNA translation as having a role in XDC (Bellodi et al., 2010b). In each case, the defect in translating these mRNAs appears connected with non-canonical modes of translation initiation. In particular, these mRNAs have been proposed to contain internal ribosome entry sites (IRES) that may recruit the ribosome to each mRNA independently of a 5′-cap. While the concept of IRES is well established in viral mRNAs, the existence of cellular IRES remains controversial (Jackson, 2013; Akirtava et al., 2022). However, defects in the synthesis of these mRNAs also explain the predisposition toward cancer observed in XDC patients.

### Williams–Beuren syndrome

Williams–Beuren syndrome (WBS) is a rare condition that affects approximately 1 in 20,000 live births and is characterized genetically by a of 1.5–1.8 Mb deletion at chromosomal position 7q11.23 (Pober, 2010). This deletion results in the loss of 28 individual genes. Patients with WBS have mild to moderate neurocognitive defects, hypersensitivity to sounds, and difficulty with visual–spatial abilities in addition to connective tissue abnormalities and premature skin aging. Because of the many genes deleted in WBS, disease symptoms are likely an accumulated effect of many insufficiencies. However, one of the 28 deleted genes encodes Nop2/Sun RNA methyltransferase 5 (NSUN5). This is a member of a family of methyltransferases that deposits methyl groups on specific RNAs, including tRNAs and rRNAs. Defects in these proteins have been associated with other neurological disorders, including WBS (Blanco et al., 2014; Paramasivam et al., 2020; Mattioli et al., 2023; PerezGrovas-Saltijeral et al., 2023).

NSUN5 is responsible for m^5^C_3782_ modification of the 28S rRNA and is the homolog of yeast Rcm1, which similarly modified m^5^C_2278_ in the yeast 25S rRNA (Sharma et al., 2013; Heissenberger et al., 2019). Loss of Rcm1 in yeast induces structural changes to the ribosome which results in a defect in translational fidelity (Schosserer et al., 2015), but also confers increased lifespan and expression of stress-responsive genes. This seeming paradox between disease state and presumed beneficial gene expression is also seen in cancer at this site (see below). Mouse knockout studies have also shown that this gene is necessary for proper cerebral cortex development, which may link the cognitive defects in WBS to the loss of this gene (Chen et al., 2019; Zhang et al., 2019). In contrast to studies in yeast, CRISPR/Cas9-mediated knockout of NSUN5 in HeLa cells decreased rates of global translation but did not show defects in translational fidelity (Heissenberger et al., 2019). Importantly, this same study formally demonstrated that the loss of one copy in WBS patients was sufficient to reduce m^5^C_3782_ levels on patient 28S rRNAs. It is currently unclear how much of the symptoms present in WBS are directly related to the haploinsufficiency of NSUN5 or a reduction of m^5^C_3782_. However, given the defects in model systems, it is worthy of further investigation and highlights the role of a single nucleotide modification in ribosome function.

### Bowen–Conradi syndrome

Bowen–Conradi syndrome (BCS) is a rare lethal autosomal recessive disorder that was first described in a pair of brothers from a Hutterite family in 1976 (Bowen and Conradi, 1976). Despite its rarity in the larger population, its incidence in Hutterite communities is greater than 1 in every 400 live births. Patients present with low birth weight, microcephaly, and anatomical malformations. Failure to thrive typically results in death within the first year of life (Hunter et al., 1979). Lineage and linkage analysis isolated the gene to a 1.9 Mbp region of chromosome 12p13.3 (Lamont et al., 2005). This region contained 59 genes. Of these, 35 were sequenced and revealed a single nucleotide change (NM_006331.6:c.400A>G) in the EMG1 gene as segregating with disease (Armistead et al., 2009). This mutation results in a D86G mutation in the EMG1 protein.

EMG1 is the human homolog of yeast Nep1, which had previously been shown to be a methyltransferase that acted upon the 18S rRNA (Eschrich et al., 2002; Buchhaupt et al., 2006). Crystallographic analysis of archaeal Nep1 had shown that D86 interacted with R84, which is essential for RNA binding (Taylor et al., 2008). Further studies of archaeal Nep1 and human EMG1 revealed that it is the *N1*-pseudouridine methyltransferase that aids in the conversion of U_1248_ to m^1^acp^3^Ψ_1248_ in the human 18S rRNA. It is also important to note that *N1*-methylation is a prerequisite for 3-amino-3-carboxypropyl modification at this location. Later, the yeast homolog of EMG1 was identified and validated to be a pseudouridine synthetase acting upon a homologous site in the yeast 18S rRNA (Meyer et al., 2011). Surprisingly, this study revealed that mutation of this aspartic acid residue did not diminish methyltransferase activity. These data strongly suggest that BCS is not a defect in rRNA modification but that EMG1 plays additional roles in the cell. Importantly, this is coupled with the initial study demonstrating that D86G results in EMG1 aggregation and a dramatic decrease in EMG1 protein in patients (Armistead et al., 2009).

Indeed, further analysis revealed that EMG1 also plays a key structural role in ribosome biogenesis. Patient samples displayed a decreased rate in 18S rRNA synthesis, although, basal levels of protein synthesis are unaffected (Armistead et al., 2014). The major phenotype thus appears to be a defect in ribosome assembly rather than in ribosome function. Indeed, siRNA-mediated depletion of EMG1 results in an accumulation of 30S precursors and a relative depletion in 18S-E precursors (Warda et al., 2016). Therefore, it is likely that BCS arises from a failure to properly form the SSU processome and efficiently generate 18S rRNA, despite the relevant mutation being found in an enzyme required for chemical modification of the rRNA.

### Alterations to rRNA modifications in cancer

The burgeoning pool of data consistently highlights significant variation in rRNA modification, particularly 2′-Ome and Ψ, during oncogenesis. Nevertheless, the prevailing trend is toward correlative connections rather than establishing clear causative effects in the majority of these instances. Misregulation of either FBL or DKC is commonly seen in cancers (Montanaro et al., 2006; Marcel et al., 2013). Unsurprisingly, this correlates with alterations in the Ψ and 2′-Ome profiles of rRNAs (Marcel et al., 2020; Barozzi et al., 2023; Zhou et al., 2023). The modulation of snoRNA expression has long been seen as a biomarker of tumorigenesis (Williams and Farzaneh, 2012; Krishnan et al., 2016; Gong et al., 2017; Barros-Silva et al., 2021). In various tumor types, decreases or increases in specific snoRNAs are common, but specific effects from gain or loss of modification guided by these snoRNAs have been more difficult to validate. Some data suggest that specific snoRNAs and the modifications they guide may promote tumor development. A form of acute myeloid leukemia (AML) undergoes genetic alterations that culminate in the enhanced formation of snoRNPs (Zhou et al., 2017). In particular, SNORD14D and SNORD35A have been shown *in vivo* and *in vitro* to be necessary for AML cells’ oncogenic properties. These two snoRNAs direct the 2′-Ome of C_1708_ in the 18S rRNA and G_1328_ in the 28S, respectively.

In contrast to Ψ and 2′-Ome modifications, there appears to be a stronger causative link between oncogenesis and base methylations. METTL5 is a methyltransferase that installs m^6^A modifications at A_1832_ of the 18S rRNA (m^6^A_1832_) (van Tran et al., 2019). Studies using mouse genetic models implicated METTL5 and m^6^A_1832_ in transcript-specific translation regulation, suggesting that METTL5 misregulation could be important in reshaping the translatome in specific disease contexts (Sepich-Poore et al., 2022). Indeed, METTL5 was shown to be upregulated in breast cancer cell lines, with its loss reducing their growth rate in vitro (Rong et al., 2020). Research suggests that m^6^A_1832_ localization to the decoding center is important for efficient translation initiation. More recently, both METTL5 and m^6^A_1832_ were found to be elevated in nasopharyngeal carcinoma (NPC) and intrahepatic cholangiocarcinoma (ICC) (Chen et al., 2023; Dai et al., 2023). Similarly, loss of METTL5 has reduced tumorigenesis *in vitro* and *in vivo* for these cancers.

Surprisingly, not all alterations to the rRNA modification profile are negative. NSUN5, which is also implicated in WBS, was identified as being alternatively regulated in a subset of gliomas (Janin et al., 2019). This gene becomes epigenetically silenced but was shown to be associated with better prognosis in patients. This same work paradoxically suggested that loss of m^5^C_3782_ reprograms the ribosomes to better translate stress-responsive mRNAs, which aids in cancer cell survival in response to tumor-associated stresses. This seeming contradiction may be reconciled by the fact that NSUN5-depleted cells have lower rates of protein synthesis. Thus, while these cells can better survive stress, they are less prolific, leading to a better prognosis for patients.

One intriguing case regarding a potential causative link occurs, again, with the case of m^1^acp^3^Ψ. Analysis of colorectal carcinoma (CRC) cells revealed downregulation of m^1^acp^3^Ψ at 1,248 of the 18S rRNA (Babaian et al., 2020). Given its location in the P-site of the ribosome, the authors suggest that the loss of this site may be key to loss of translational control seen during oncogenesis. Indeed, loss of the acp^3^ moiety following TSR3 knockout revealed an increase in ribosomal protein synthesis, an important driver in cancer development. While more work is needed to confirm loss of m^1^acp^3^Ψ as an oncogenic driver, these data support the hypothesis that alterations to individual modifications can promote cancer development.

## Figures and Tables

**TABLE 1:** Evolutionary conservation of ribosomal components. Ribosomal proteins and rRNAs found in *Homo sapiens*, *S. cerevisiae*, and *E. coli*. Note that the lengths of each mature rRNA are an approximation as there is heterogeneity in rRNA length amongst individuals or between strains. Numbers included here are derived from indicated GenBank or RefSeq submissions.

	Ribosomal Proteins			Ribosomal RNA Species		
	Large	Small	Total	Precursor	Name	Length (nt)
*H. Sapiens*	47	33	80	Pre-5S	5S	119 (XR_007071486.1)
				Tricistronic 47S pre-rRNA	18S	1869 (XR_007090847.1)
					5.8S	151 (XR_007090886.1)
					28S	5071 (XR_007090869.1)
*S. cerevisiae*	46	33	79	Pre-5S	5S	121 (NC_001144.5)
				Tricistronic 35S pre-rRNA	18S	1730 (NG_063315.1)
					5.8S	158 (NR_132220.1)
					25S	3396 (NR_132218.1)
*E. coli*	33	22	55		16S	1450 (NR_024570.1)
					23S	2905 (NR_076322.1)
					5S	120 (NR_075284.1)

**TABLE 2:** Universally conserved residues in rRNA. Positions of residues within the decoding center and peptidyl transferase domains in *H. sapiens*, *S. cerevisiae*, and *E. coli*. As with the lengths, note that the exact location of these sites may be different in an individual molecule due to sequence heterogeneity. However, the identity of these residues does not vary.

	Decoding center (DC) key conserved residues		
18S/16S	*H. sapiens*	*S. cerevisiae*	*E. coli*
	G626	G567	G530
	A1824	A1781	A1492
	A1825	A1782	A1493
	Peptidyl transferase center (PTC) key conserved residues		
18S/16S	*H. sapiens*	*S. cerevisiae*	*E. coli*
	G1693	G1575	G1338
	A1640	A1576	A1339

## References

[R1] Aalfs CM, van den Berg H, Barth PG, and Hennekam RC (1995). The Hoyeraal-Hreidarsson syndrome: the fourth case of a separate entity with prenatal growth retardation, progressive pancytopenia and cerebellar hypoplasia. Eur. J. Pediatr 154, 304–308. doi:10.1007/bf01957367 7607282

[R2] Akirtava C, May GE, and McManus CJ (2022). False-positive IRESes from Hoxa9 and other genes resulting from errors in mammalian 5’ UTR annotations. Proc. Natl. Acad. Sci. U. S. A 119, e2122170119. doi:10.1073/pnas.2122170119 36037358 PMC9456764

[R3] Allmang C, Petfalski E, Podtelejnikov A, Mann M, Tollervey D, and Mitchell P (1999). The yeast exosome and human PM-Scl are related complexes of 3’ right-arrow 5’ exonucleases. Genes & Dev. 13, 2148–2158. doi:10.1101/gad.13.16.2148 10465791 PMC316947

[R4] Alupei MC, Maity P, Esser PR, Krikki I, Tuorto F, Parlato R, (2018). Loss of proteostasis is a pathomechanism in cockayne syndrome. Cell Rep. 23, 1612–1619. doi:10.1016/j.celrep.2018.04.041 29742419

[R5] Amberg DC, Goldstein AL, and Cole CN (1992). Isolation and characterization of RAT1: an essential gene of *Saccharomyces cerevisiae* required for the efficient nucleocytoplasmic trafficking of mRNA. Genes Dev. 6, 1173–1189. doi:10.1101/gad.6.7.1173 1628825

[R6] Ameismeier M, Zemp I, van den Heuvel J, Thoms M, Berninghausen O, Kutay U, (2020). Structural basis for the final steps of human 40S ribosome maturation. Nature 587, 683–687. doi:10.1038/s41586-020-2929-x 33208940

[R7] Anger AM, Armache JP, Berninghausen O, Habeck M, Subklewe M, Wilson DN, (2013). Structures of the human and Drosophila 80S ribosome. Nature 497, 80–85. doi:10.1038/nature12104 23636399

[R8] Ansel KM, Pastor WA, Rath N, Lapan AD, Glasmacher E, Wolf C, (2008). Mouse Eri1 interacts with the ribosome and catalyzes 5.8S rRNA processing. Nat. Struct. Mol. Biol 15, 523–530. doi:10.1038/nsmb.1417 18438418 PMC3032500

[R9] Arabi A, Wu S, Ridderstrale K, Bierhoff H, Shiue C, Fatyol K, (2005). c-Myc associates with ribosomal DNA and activates RNA polymerase I transcription. Nat. Cell Biol 7, 303–310. doi:10.1038/ncb1225 15723053

[R10] Arimbasseri AG, Rijal K, and Maraia RJ (2013). Transcription termination by the eukaryotic RNA polymerase III. Biochimica Biophysica Acta (BBA) - Gene Regul. Mech 1829, 318–330. doi:10.1016/j.bbagrm.2012.10.006 PMC3568203 23099421

[R11] Armistead J, Hemming R, Patel N, and Triggs-Raine B (2014). Mutation of EMG1 causing Bowen-Conradi syndrome results in reduced cell proliferation rates concomitant with G2/M arrest and 18S rRNA processing delay. BBA Clin. 1, 33–43. doi:10.1016/j.bbacli.2014.05.002 26676230 PMC4633970

[R12] Armistead J, Khatkar S, Meyer B, Mark BL, Patel N, Coghlan G, (2009). Mutation of a gene essential for ribosome biogenesis, EMG1, causes Bowen-Conradi syndrome. Am. J. Hum. Genet 84, 728–739. doi:10.1016/j.ajhg.2009.04.017 19463982 PMC2694972

[R13] Assfalg R, Lebedev A, Gonzalez OG, Schelling A, Koch S, and Iben S (2012). TFIIH is an elongation factor of RNA polymerase I. Nucleic Acids Res. 40, 650–659. doi:10.1093/nar/gkr746 21965540 PMC3258137

[R14] Babaian A, Rothe K, Girodat D, Minia I, Djondovic S, Milek M, (2020). Loss of m1acp3Ψ ribosomal RNA modification is a major feature of cancer. Cell Rep. 31, 107611. doi:10.1016/j.celrep.2020.107611 32375039

[R15] Bachellerie JP, Michot B, and Raynal F (1983). Recognition signals for mouse pre-rRNA processing. A potential role for U3 nucleolar RNA. Mol. Biol. Rep 9, 79–86. doi:10.1007/bf00777477 6193412

[R16] Balogh E, Chandler JC, Varga M, Tahoun M, Menyhard DK, Schay G, (2020). Pseudouridylation defect due to DKC1 and NOP10 mutations causes nephrotic syndrome with cataracts, hearing impairment, and enterocolitis. Proc. Natl. Acad. Sci. U. S. A 117, 15137–15147. doi:10.1073/pnas.2002328117 32554502 PMC7334496

[R17] Ban N, Nissen P, Hansen J, Moore PB, and Steitz TA (2000). The complete atomic structure of the large ribosomal subunit at 2.4 A resolution. Science 289, 905–920. doi:10.1126/science.289.5481.905 10937989

[R18] Barozzi C, Zacchini F, Corradini AG, Morara M, Serra M, De Sanctis V, (2023). Alterations of ribosomal RNA pseudouridylation in human breast cancer. Nar. Cancer 5, zcad026. doi:10.1093/narcan/zcad026 37260601 PMC10227372

[R19] Barros-Silva D, Klavert J, Jenster G, Jeronimo C, Lafontaine DLJ, and Martens-Uzunova ES (2021). The role of OncoSnoRNAs and ribosomal RNA 2’-O-methylation in cancer. RNA Biol. 18, 61–74. doi:10.1080/15476286.2021.1991167 34775914 PMC8677010

[R20] Barta A, Steiner G, Brosius J, Noller HF, and Kuechler E (1984). Identification of a site on 23S ribosomal RNA located at the peptidyl transferase center. Proc. Natl. Acad. Sci. U. S. A 81, 3607–3611. doi:10.1073/pnas.81.12.3607 6374660 PMC345267

[R21] Beckmann H, Chen JL, O’Brien T, and Tjian R (1995). Coactivator and promoter-selective properties of RNA polymerase I TAFs. Science 270, 1506–1509. doi:10.1126/science.270.5241.1506 7491500

[R22] Bell SP, Learned RM, Jantzen HM, and Tjian R (1988). Functional cooperativity between transcription factors UBF1 and SL1 mediates human ribosomal RNA synthesis. Science 241, 1192–1197. doi:10.1126/science.3413483 3413483

[R23] Bellodi C, Kopmar N, and Ruggero D (2010b). Deregulation of oncogene-induced senescence and p53 translational control in X-linked dyskeratosis congenita. EMBO J. 29, 1865–1876. doi:10.1038/emboj.2010.83 20453831 PMC2885924

[R24] Bellodi C, Krasnykh O, Haynes N, Theodoropoulou M, Peng G, Montanaro L, (2010a). Loss of function of the tumor suppressor DKC1 perturbs p27 translation control and contributes to pituitary tumorigenesis. Cancer Res. 70, 6026–6035. doi:10.1158/0008-5472.can-09-4730 20587522 PMC2913864

[R25] Ben-Shem A, Garreau de Loubresse N, Melnikov S, Jenner L, Yusupova G, and Yusupov M (2011). The structure of the eukaryotic ribosome at 3.0 A resolution. Science 334, 1524–1529. doi:10.1126/science.1212642 22096102

[R26] Ben-Shem A, Jenner L, Yusupova G, and Yusupov M (2010). Crystal structure of the eukaryotic ribosome. Science 330, 1203–1209. doi:10.1126/science.1194294 21109664

[R27] Benyelles M, O’Donohue MF, Kermasson L, Lainey E, Borie R, Lagresle-Peyrou C, (2020). NHP2 deficiency impairs rRNA biogenesis and causes pulmonary fibrosis and Hoyeraal-Hreidarsson syndrome. Hum. Mol. Genet 29, 907–922. doi:10.1093/hmg/ddaa011 31985013

[R28] Betard C, Rasquin-Weber A, Brewer C, Drouin E, Clark S, Verner A, (2000). Localization of a recessive gene for North American Indian childhood cirrhosis to chromosome region 16q22-and identification of a shared haplotype. Am. J. Hum. Genet 67, 222–228. doi:10.1086/302993 10820129 PMC1287080

[R29] Birkedal U, Christensen-Dalsgaard M, Krogh N, Sabarinathan R, Gorodkin J, and Nielsen H (2015). Profiling of ribose methylations in RNA by high-throughput sequencing. Angew. Chem. Int. Ed 54, 451–455. doi:10.1002/anie.201408362 25417815

[R30] Blanco S, Dietmann S, Flores JV, Hussain S, Kutter C, Humphreys P, (2014). Aberrant methylation of tRNAs links cellular stress to neuro-developmental disorders. EMBO J. 33, 2020–2039. doi:10.15252/embj.201489282 25063673 PMC4195770

[R31] Blattner C, Jennebach S, Herzog F, Mayer A, Cheung AC, Witte G, (2011). Molecular basis of Rrn3-regulated RNA polymerase I initiation and cell growth. Genes Dev. 25, 2093–2105. doi:10.1101/gad.17363311 21940764 PMC3197207

[R32] Bleichert F, Granneman S, Osheim YN, Beyer AL, and Baserga SJ (2006). The PINc domain protein Utp24, a putative nuclease, is required for the early cleavage steps in 18S rRNA maturation. Proc. Natl. Acad. Sci. U. S. A 103, 9464–9469. doi:10.1073/pnas.0603673103 16769905 PMC1480430

[R33] Bodem J, Dobreva G, Hoffmann-Rohrer U, Iben S, Zentgraf H, Delius H, (2000). TIF-IA, the factor mediating growth-dependent control of ribosomal RNA synthesis, is the mammalian homolog of yeast Rrn3p. EMBO Rep. 1, 171–175. doi:10.1093/embo-reports/kvd032 11265758 PMC1084264

[R34] Bowen P, and Conradi GJ (1976). Syndrome of skeletal and genitourinary anomalies with unusual facies and failure to thrive in Hutterite sibs. Birth Defects Orig. Artic. Ser 12, 101–108. 974244

[R35] Bradsher J, Auriol J, de Santis LP, Iben S, Vonesch JL, Grummt I, (2002). CSB is a component of RNA pol I transcription. Mol. Cell 10, 819–829. doi:10.1016/s1097-2765(02)00678-0 12419226

[R36] Brouwer R, Allmang C, Raijmakers R, van Aarssen Y, Egberts WV, Petfalski E, (2001). Three novel components of the human exosome. J. Biol. Chem 276, 6177–6184. doi:10.1074/jbc.m007603200 11110791

[R37] Brun RP, Ryan K, and Sollner-Webb B (1994). Factor C*, the specific initiation component of the mouse RNA polymerase I holoenzyme, is inactivated early in the transcription process. Mol. Cell. Biol 14, 5010–5021. doi:10.1128/mcb.14.7.5010-5021.1994 8007994 PMC358872

[R38] Bruno PM, Lu M, Dennis KA, Inam H, Moore CJ, Sheehe J, (2020). The primary mechanism of cytotoxicity of the chemotherapeutic agent CX-5461 is topoisomerase II poisoning. Proc. Natl. Acad. Sci. U. S. A 117, 4053–4060. doi:10.1073/pnas.1921649117 32041867 PMC7049172

[R39] Buchhaupt M, Meyer B, Kotter P, and Entian KD (2006). Genetic evidence for 18S rRNA binding and an Rps19p assembly function of yeast nucleolar protein Nep1p. Mol. Genet. Genomics 276, 273–284. doi:10.1007/s00438-006-0132-x 16721597

[R40] Budkevich TV, Giesebrecht J, Behrmann E, Loerke J, Ramrath DJ, Mielke T, (2014). Regulation of the mammalian elongation cycle by subunit rolling: a eukaryotic-specific ribosome rearrangement. Cell 158, 121–131. doi:10.1016/j.cell.2014.04.044 24995983 PMC4141720

[R41] Buttgereit D, Pflugfelder G, and Grummt I (1985). Growth-dependent regulation of rRNA synthesis is mediated by a transcription initiation factor (TTF-IA). Nucleic Acids Res. 13, 8165–8180. doi:10.1093/nar/13.22.8165 4070001 PMC322117

[R42] Caburet S, Conti C, Schurra C, Lebofsky R, Edelstein SJ, and Bensimon A (2005). Human ribosomal RNA gene arrays display a broad range of palindromic structures. Genome Res. 15, 1079–1085. doi:10.1101/gr.3970105 16024823 PMC1182220

[R43] Cadwell C, Yoon HJ, Zebarjadian Y, and Carbon J (1997). The yeast nucleolar protein Cbf5p is involved in rRNA biosynthesis and interacts genetically with the RNA polymerase I transcription factor RRN3. Mol. Cell. Biol 17, 6175–6183. doi:10.1128/mcb.17.10.6175 9315678 PMC232468

[R44] Calvet JP, and Pederson T (1981). Base-pairing interactions between small nuclear RNAs and nuclear RNA precursors as revealed by psoralen cross-linking *in vivo*. Cell 26, 363–370. doi:10.1016/0092-8674(81)90205-1 6173132

[R45] Carron C, O’Donohue MF, Choesmel V, Faubladier M, and Gleizes PE (2011). Analysis of two human pre-ribosomal factors, bystin and hTsr1, highlights differences in evolution of ribosome biogenesis between yeast and mammals. Nucleic Acids Res. 39, 280–291. doi:10.1093/nar/gkq734 20805244 PMC3017594

[R46] Castle CD, Cassimere EK, and Denicourt C (2012). LAS1L interacts with the mammalian Rix1 complex to regulate ribosome biogenesis. Mol. Biol. Cell 23, 716–728. doi:10.1091/mbc.e11-06-0530 22190735 PMC3279398

[R47] Castle CD, Cassimere EK, Lee J, and Denicourt C (2010). Las1L is a nucleolar protein required for cell proliferation and ribosome biogenesis. Mol. Cell. Biol 30, 4404–4414. doi:10.1128/mcb.00358-10 20647540 PMC2937536

[R48] Chagnon P, Michaud J, Mitchell G, Mercier J, Marion JF, Drouin E, (2002). A missense mutation (R565W) in cirhin (FLJ14728) in North American Indian childhood cirrhosis. Am. J. Hum. Genet 71, 1443–1449. doi:10.1086/344580 12417987 PMC378590

[R49] Chang DD, and Clayton DA (1987a). A novel endoribonuclease cleaves at a priming site of mouse mitochondrial DNA replication. EMBO J. 6, 409–417. doi:10.1002/j.1460-2075.1987.tb04770.x 3582365 PMC553411

[R50] Chang DD, and Clayton DA (1987b). A mammalian mitochondrial RNA processing activity contains nucleus-encoded RNA. Science 235, 1178–1184. doi:10.1126/science.2434997 2434997

[R51] Chen B, Huang Y, He S, Yu P, Wu L, and Peng H (2023). N(6)-methyladenosine modification in 18S rRNA promotes tumorigenesis and chemoresistance via HSF4b/HSP90B1/mutant p53 axis. Cell Chem. Biol 30, 144–158.e10. doi:10.1016/j.chembiol.2023.01.006 36800991

[R52] Chen P, Zhang T, Yuan Z, Shen B, and Chen L (2019). Expression of the RNA methyltransferase Nsun5 is essential for developing cerebral cortex. Mol. Brain 12, 74. doi:10.1186/s13041-019-0496-6 31462248 PMC6714381

[R53] Choucair N, Rajab M, Megarbane A, and Chouery E (2017). Homozygous microdeletion of the ERI1 and MFHAS1 genes in a patient with intellectual disability, limb abnormalities, and cardiac malformation. Am. J. Med. Genet. Part A 173, 1955–1960. doi:10.1002/ajmg.a.38271 28488351

[R54] Chu S, Archer RH, Zengel JM, and Lindahl L (1994). The RNA of RNase MRP is required for normal processing of ribosomal RNA. Proc. Natl. Acad. Sci. U. S. A 91, 659–663. doi:10.1073/pnas.91.2.659 8290578 PMC43008

[R55] Cochella L, Brunelle JL, and Green R (2007). Mutational analysis reveals two independent molecular requirements during transfer RNA selection on the ribosome. Nat. Struct. Mol. Biol 14, 30–36. doi:10.1038/nsmb1183 17159993

[R56] Comai L, Tanese N, and Tjian R (1992). The TATA-binding protein and associated factors are integral components of the RNA polymerase I transcription factor, SL1. Cell 68, 965–976. doi:10.1016/0092-8674(92)90039-f 1547496

[R57] Comai L, Zomerdijk JC, Beckmann H, Zhou S, Admon A, and Tjian R (1994). Reconstitution of transcription factor SL1: exclusive binding of TBP by SL1 or TFIID subunits. Science 266, 1966–1972. doi:10.1126/science.7801123 7801123

[R58] Connor JM, Gatherer D, Gray FC, Pirrit LA, and Affara NA (1986). Assignment of the gene for dyskeratosis congenita to Xq28. Hum. Genet 72, 348–351. doi:10.1007/bf00290963 3009302

[R59] Correll CC, Munishkin A, Chan YL, Ren Z, Wool IG, and Steitz TA (1998). Crystal structure of the ribosomal RNA domain essential for binding elongation factors. Proc. Natl. Acad. Sci. U. S. A 95, 13436–13441. doi:10.1073/pnas.95.23.13436 9811818 PMC24837

[R60] Coute Y, Kindbeiter K, Belin S, Dieckmann R, Duret L, Bezin L, (2008). ISG20L2, a novel vertebrate nucleolar exoribonuclease involved in ribosome biogenesis. Mol. Cell. Proteomics 7, 546–559. doi:10.1074/mcp.m700510-mcp200 18065403

[R61] Crouch RJ, Kanaya S, and Earl PL (1983). A model for the involvement of the small nucleolar RNA (U3) in processing eukaryotic ribosomal RNA. Mol. Biol. Rep 9, 75–78. doi:10.1007/bf00777476 6193411

[R62] Dai Z, Zhu W, Hou Y, Zhang X, Ren X, Lei K, (2023). METTL5-mediated 18S rRNA m(6)A modification promotes oncogenic mRNA translation and intrahepatic cholangiocarcinoma progression. Mol. Ther 31, 3225–3242. doi:10.1016/j.ymthe.2023.09.014 37735874 PMC10638452

[R63] Dammann R, Lucchini R, Koller T, and Sogo JM (1995). Transcription in the yeast rRNA gene locus: distribution of the active gene copies and chromatin structure of their flanking regulatory sequences. Mol. Cell. Biol 15, 5294–5303. doi:10.1128/mcb.15.10.5294 7565678 PMC230777

[R64] Dauwerse JG, Dixon J, Seland S, Ruivenkamp CA, van Haeringen A, Hoefsloot LH, (2011). Mutations in genes encoding subunits of RNA polymerases I and III cause Treacher Collins syndrome. Nat. Genet 43, 20–22. doi:10.1038/ng.724 21131976

[R65] Dixon J, Edwards SJ, Gladwin AJ, Dixon MJ, Loftus SK, Bonner CA, (1996). Positional cloning of a gene involved in the pathogenesis of Treacher Collins syndrome. Nat. Genet 12, 130–136. doi:10.1038/ng0296-130 8563749

[R66] Dixon J, Jones NC, Sandell LL, Jayasinghe SM, Crane J, Rey JP, (2006). Tcof1/Treacle is required for neural crest cell formation and proliferation deficiencies that cause craniofacial abnormalities. Proc. Natl. Acad. Sci. U. S. A 103, 13403–13408. doi:10.1073/pnas.0603730103 16938878 PMC1557391

[R67] Dominski Z, Yang XC, Kaygun H, Dadlez M, and Marzluff WF (2003). A 3’ exonuclease that specifically interacts with the 3’ end of histone mRNA. Mol. Cell 12, 295–305. doi:10.1016/s1097-2765(03)00278-8 14536070

[R68] Dragon F, Gallagher JE, Compagnone-Post PA, Mitchell BM, Porwancher KA, Wehner KA, (2002). A large nucleolar U3 ribonucleoprotein required for 18S ribosomal RNA biogenesis. Nature 417, 967–970. doi:10.1038/nature00769 12068309 PMC11487672

[R69] Drygin D, Siddiqui-Jain A, O’Brien S, Schwaebe M, Lin A, Bliesath J, (2009). Anticancer activity of CX-3543: a direct inhibitor of rRNA biogenesis. Cancer Res. 69, 7653–7661. doi:10.1158/0008-5472.can-09-1304 19738048

[R70] Duployez N, Vasseur L, Kim R, Largeaud L, Passet M, L’Haridon A, (2023). UBTF tandem duplications define a distinct subtype of adult *de novo* acute myeloid leukemia. Leukemia 37, 1245–1253. doi:10.1038/s41375-023-01906-z 37085611 PMC10244165

[R71] Emerson CP (1971). Regulation of the synthesis and the stability of ribosomal RNA during contact inhibition of growth. Nat. New Biol 232, 101–106. doi:10.1038/newbio232101a0 5284943

[R72] Erlacher MD, Lang K, Shankaran N, Wotzel B, Huttenhofer A, Micura R, (2005). Chemical engineering of the peptidyl transferase center reveals an important role of the 2’-hydroxyl group of A2451. Nucleic Acids Res. 33, 1618–1627. doi:10.1093/nar/gki308 15767286 PMC1065261

[R73] Eschrich D, Buchhaupt M, Kotter P, and Entian KD (2002). Nep1p (Emg1p), a novel protein conserved in eukaryotes and archaea, is involved in ribosome biogenesis. Curr. Genet 40, 326–338. doi:10.1007/s00294-001-0269-4 11935223

[R74] Fay MM, Lyons SM, and Ivanov P (2017). RNA G-quadruplexes in biology: principles and molecular mechanisms. J. Mol. Biol 429, 2127–2147. doi:10.1016/j.jmb.2017.05.017 28554731 PMC5603239

[R75] Ferretti MB, and Karbstein K (2019). Does functional specialization of ribosomes really exist? RNA 25, 521–538. doi:10.1261/rna.069823.118 30733326 PMC6467006

[R76] Freed EF, Prieto JL, McCann KL, McStay B, and Baserga SJ (2012). NOL11, implicated in the pathogenesis of North American Indian childhood cirrhosis, is required for pre-rRNA transcription and processing. PLoS Genet. 8, e1002892. doi:10.1371/journal.pgen.1002892 22916032 PMC3420923

[R77] Friedrich JK, Panov KI, Cabart P, Russell J, and Zomerdijk JC (2005). TBP-TAF complex SL1 directs RNA polymerase I pre-initiation complex formation and stabilizes upstream binding factor at the rDNA promoter. J. Biol. Chem 280, 29551–29558. doi:10.1074/jbc.m501595200 15970593 PMC3858828

[R78] Fromm L, Falk S, Flemming D, Schuller JM, Thoms M, Conti E, (2017). Reconstitution of the complete pathway of ITS2 processing at the pre-ribosome. Nat. Commun 8, 1787. doi:10.1038/s41467-017-01786-9 29176610 PMC5702609

[R79] Gabel HW, and Ruvkun G (2008). The exonuclease ERI-1 has a conserved dual role in 5.8S rRNA processing and RNAi. Nat. Struct. Mol. Biol 15, 531–533. doi:10.1038/nsmb.1411 18438419 PMC2910399

[R80] Gallagher JE, Dunbar DA, Granneman S, Mitchell BM, Osheim Y, Beyer AL, (2004). RNA polymerase I transcription and pre-rRNA processing are linked by specific SSU processome components. Genes Dev. 18, 2506–2517. doi:10.1101/gad.1226604 15489292 PMC529538

[R81] Gasse L, Flemming D, and Hurt E (2015). Coordinated ribosomal ITS2 RNA processing by the Las1 complex integrating endonuclease, polynucleotide kinase, and exonuclease activities. Mol. Cell 60, 808–815. doi:10.1016/j.molcel.2015.10.021 26638174

[R82] Gaubatz J, Prashad N, and Cutler RG (1976). Ribosomal RNA gene dosage as a function of tissue and age for mouse and human. Biochimica Biophysica Acta (BBA) - Nucleic Acids Protein Synthesis 418, 358–375. doi:10.1016/0005-2787(76)90297-5 1247550

[R83] Geerlings TH, Vos JC, and Raue HA (2000). The final step in the formation of 25S rRNA in *Saccharomyces cerevisiae* is performed by 5’-->3’ exonucleases. RNA 6, 1698–1703. doi:10.1017/s1355838200001540 11142370 PMC1370040

[R84] Genuth NR, and Barna M (2018). The discovery of ribosome heterogeneity and its implications for gene regulation and organismal life. Mol. Cell 71, 364–374. doi:10.1016/j.molcel.2018.07.018 30075139 PMC6092941

[R85] Gerstberger S, Meyer C, Benjamin-Hong S, Rodriguez J, Briskin D, Bognanni C, (2017). The conserved RNA exonuclease Rexo5 is required for 3’ end maturation of 28S rRNA, 5S rRNA, and snoRNAs. Cell Rep. 21, 758–772. doi:10.1016/j.celrep.2017.09.067 29045842 PMC5662206

[R86] Goldfarb KC, and Cech TR (2017). Targeted CRISPR disruption reveals a role for RNase MRP RNA in human preribosomal RNA processing. Genes Dev. 31, 59–71. doi:10.1101/gad.286963.116 28115465 PMC5287113

[R87] Gong J, Li Y, Liu CJ, Xiang Y, Li C, Ye Y, (2017). A pan-cancer analysis of the expression and clinical relevance of small nucleolar RNAs in human cancer. Cell Rep. 21, 1968–1981. doi:10.1016/j.celrep.2017.10.070 29141226

[R88] Gonzales B, Henning D, So RB, Dixon J, Dixon MJ, and Valdez BC (2005). The Treacher Collins syndrome (TCOF1) gene product is involved in pre-rRNA methylation. Hum. Mol. Genet 14, 2035–2043. doi:10.1093/hmg/ddi208 15930015

[R89] Gorlin RJ, Cohen MM, and Levin LS (1990). Syndromes of the head and neck. New York: Oxford University Press.

[R90] Gorski JJ, Pathak S, Panov K, Kasciukovic T, Panova T, Russell J, (2007). A novel TBP-associated factor of SL1 functions in RNA polymerase I transcription. EMBO J. 26, 1560–1568. doi:10.1038/sj.emboj.7601601 17318177 PMC1829371

[R91] Grandori C, Gomez-Roman N, Felton-Edkins ZA, Ngouenet C, Galloway DA, Eisenman RN, (2005). c-Myc binds to human ribosomal DNA and stimulates transcription of rRNA genes by RNA polymerase I. Nat. Cell Biol 7, 311–318. doi:10.1038/ncb1224 15723054

[R92] Grewal SS, Li L, Orian A, Eisenman RN, and Edgar BA (2005). Myc-dependent regulation of ribosomal RNA synthesis during Drosophila development. Nat. Cell Biol 7, 295–302. doi:10.1038/ncb1223 15723055

[R93] Grummt I (1982). Nucleotide sequence requirements for specific initiation of transcription by RNA polymerase I. Proc. Natl. Acad. Sci. U. S. A 79, 6908–6911. doi:10.1073/pnas.79.22.6908 6294665 PMC347243

[R94] Grummt I, Roth E, and Paule MR (1982). Ribosomal RNA transcription *in vitro* is species specific. Nature 296, 173–174. doi:10.1038/296173a0 7063022

[R95] Gu BW, Apicella M, Mills J, Fan JM, Reeves DA, French D, (2015). Impaired telomere maintenance and decreased canonical WNT signaling but normal ribosome biogenesis in induced pluripotent stem cells from X-linked dyskeratosis congenita patients. PLoS One 10, e0127414. doi:10.1371/journal.pone.0127414 25992652 PMC4436374

[R96] Guo L, Salian S, Xue JY, Rath N, Rousseau J, Kim H, (2023). Null and missense mutations of ERI1 cause a recessive phenotypic dichotomy in humans. Am. J. Hum. Genet 110, 1068–1085. doi:10.1016/j.ajhg.2023.06.001 37352860 PMC10357479

[R97] Hafner SJ, Jansson MD, Altinel K, Andersen KL, Abay-Norgaard Z, Menard P, (2023). Ribosomal RNA 2’-O-methylation dynamics impact cell fate decisions. Dev. Cell 58, 1593–1609.e9. doi:10.1016/j.devcel.2023.06.007 37473757

[R98] Hallgren J, Pietrzak M, Rempala G, Nelson PT, and Hetman M (2014). Neurodegeneration-associated instability of ribosomal DNA. Biochimica Biophysica Acta (BBA) - Mol. Basis Dis 1842, 860–868. doi:10.1016/j.bbadis.2013.12.012 PMC3985612 24389328

[R99] Hamada H, Muramatsu M, Urano Y, Onishi T, and Kominami R (1979). *In vitro* synthesis of a 5S RNA precursor by isolated nuclei of rat liver and HeLa cells. Cell 17, 163–173. doi:10.1016/0092-8674(79)90304-0 110459

[R100] Hampl H, Schulze H, and Nierhaus KH (1981). Ribosomal components from *Escherichia coli* 50 S subunits involved in the reconstitution of peptidyltransferase activity. J. Biol. Chem 256, 2284–2288. doi:10.1016/s0021-9258(19)69775-9 7007380

[R101] Hayano T, Yanagida M, Yamauchi Y, Shinkawa T, Isobe T, and Takahashi N (2003). Proteomic analysis of human nop56p-associated pre-ribosomal ribonucleoprotein complexes. J. Biol. Chem 278, 34309–34319. doi:10.1074/jbc.m304304200 12777385

[R102] Hebras J, Krogh N, Marty V, Nielsen H, and Cavaille J (2020). Developmental changes of rRNA ribose methylations in the mouse. RNA Biol. 17, 150–164. doi:10.1080/15476286.2019.1670598 31566069 PMC6948968

[R103] Heindl K, and Martinez J (2010). Nol9 is a novel polynucleotide 5’-kinase involved in ribosomal RNA processing. EMBO J. 29, 4161–4171. doi:10.1038/emboj.2010.275 21063389 PMC3018789

[R104] Heiss NS, Knight SW, Vulliamy TJ, Klauck SM, Wiemann S, Mason PJ, (1998). X-linked dyskeratosis congenita is caused by mutations in a highly conserved gene with putative nucleolar functions. Nat. Genet 19, 32–38. doi:10.1038/ng0598-32 9590285

[R105] Heissenberger C, Liendl L, Nagelreiter F, Gonskikh Y, Yang G, Stelzer EM, (2019). Loss of the ribosomal RNA methyltransferase NSUN5 impairs global protein synthesis and normal growth. Nucleic Acids Res. 47, 11807–11825. doi:10.1093/nar/gkz1043 31722427 PMC7145617

[R106] Heix J, Zomerdijk JC, Ravanpay A, Tjian R, and Grummt I (1997). Cloning of murine RNA polymerase I-specific TAF factors: conserved interactions between the subunits of the species-specific transcription initiation factor TIF-IB/SL1. Proc. Natl. Acad. Sci. U. S. A 94, 1733–1738. doi:10.1073/pnas.94.5.1733 9050847 PMC19985

[R107] Henderson AS, Warburton D, and Atwood KC (1972). Location of ribosomal DNA in the human chromosome complement. Proc. Natl. Acad. Sci. U. S. A 69, 3394–3398. doi:10.1073/pnas.69.11.3394 4508329 PMC389778

[R108] Henning KA, Li L, Iyer N, McDaniel LD, Reagan MS, Legerski R, (1995). The Cockayne syndrome group A gene encodes a WD repeat protein that interacts with CSB protein and a subunit of RNA polymerase II TFIIH. Cell 82, 555–564. doi:10.1016/0092-8674(95)90028-4 7664335

[R109] Henry Y, Wood H, Morrissey JP, Petfalski E, Kearsey S, and Tollervey D (1994). The 5’ end of yeast 5.8S rRNA is generated by exonucleases from an upstream cleavage site. EMBO J. 13, 2452–2463. doi:10.1002/j.1460-2075.1994.tb06530.x 7515008 PMC395111

[R110] Hirschler-Laszkiewicz I, Cavanaugh AH, Mirza A, Lun M, Hu Q, Smink T, (2003). Rrn3 becomes inactivated in the process of ribosomal DNA transcription. J. Biol. Chem 278, 18953–18959. doi:10.1074/jbc.m301093200 12646563

[R111] Hoareau-Aveilla C, Fayet-Lebaron E, Jady BE, Henras AK, and Kiss T (2012). Utp23p is required for dissociation of snR30 small nucleolar RNP from preribosomal particles. Nucleic Acids Res. 40, 3641–3652. doi:10.1093/nar/gkr1213 22180534 PMC3333846

[R112] Holm M, Natchiar SK, Rundlet EJ, Myasnikov AG, Watson ZL, Altman RB, (2023). mRNA decoding in human is kinetically and structurally distinct from bacteria. Nature 617, 200–207. doi:10.1038/s41586-023-05908-w 37020024 PMC10156603

[R113] Hoppe S, Bierhoff H, Cado I, Weber A, Tiebe M, Grummt I, (2009). AMP-activated protein kinase adapts rRNA synthesis to cellular energy supply. Proc. Natl. Acad. Sci. U. S. A 106, 17781–17786. doi:10.1073/pnas.0909873106 19815529 PMC2764937

[R114] Hotz M, Thayer NH, Hendrickson DG, Schinski EL, Xu J, and Gottschling DE (2022). rDNA array length is a major determinant of replicative lifespan in budding yeast. Proc. Natl. Acad. Sci. U. S. A 119, e2119593119. doi:10.1073/pnas.2119593119 35394872 PMC9169770

[R115] Hoxha V, and Aliu E (2023). ERI1: a case report of an autosomal recessive syndrome associated with developmental delay and distal limb abnormalities. Am. J. Med. Genet. Part A 191, 64–69. doi:10.1002/ajmg.a.62987 36208065

[R116] Hoyeraal HM, Lamvik J, and Moe PJ (1970). Congenital hypoplastic thrombocytopenia and cerebral malformations in two brothers. Acta Paediatr. 59, 185–191. doi:10.1111/j.1651-2227.1970.tb08986.x 5442429

[R117] Hunter AG, Woerner SJ, Montalvo-Hicks LD, Fowlow SB, Haslam RH, Metcalf PJ, (1979). The Bowen-Conradi syndrome – a highly lethal autosomal recessive syndrome of microcephaly, micrognathia, low birth weight, and joint deformities. Am. J. Med. Genet 3, 269–279. doi:10.1002/ajmg.1320030305 484596

[R118] Iben S, Tschochner H, Bier M, Hoogstraten D, Hozak P, Egly JM, (2002). TFIIH plays an essential role in RNA polymerase I transcription. Cell 109, 297–306. doi:10.1016/s0092-8674(02)00729-8 12015980

[R119] Iida T, Kawaguchi R, and Nakayama J (2006). Conserved ribonuclease, Eri1, negatively regulates heterochromatin assembly in fission yeast. Curr. Biol 16, 1459–1464. doi:10.1016/j.cub.2006.05.061 16797182

[R120] Isaac C, Marsh KL, Paznekas WA, Dixon J, Dixon MJ, Jabs EW, (2000). Characterization of the nucleolar gene product, treacle, in Treacher Collins syndrome. Mol. Biol. Cell 11, 3061–3071. doi:10.1091/mbc.11.9.3061 10982400 PMC14975

[R121] Ito S, Akamatsu Y, Noma A, Kimura S, Miyauchi K, Ikeuchi Y, (2014a). A single acetylation of 18 S rRNA is essential for biogenesis of the small ribosomal subunit in *Saccharomyces cerevisiae*. J. Biol. Chem 289, 26201–26212. doi:10.1074/jbc.m114.593996 25086048 PMC4176211

[R122] Ito S, Horikawa S, Suzuki T, Kawauchi H, Tanaka Y, Suzuki T, (2014b). Human NAT10 is an ATP-dependent RNA acetyltransferase responsible for N4-acetylcytidine formation in 18 S ribosomal RNA (rRNA). J. Biol. Chem 289, 35724–35730. doi:10.1074/jbc.c114.602698 25411247 PMC4276842

[R123] Jackson RJ (2013). The current status of vertebrate cellular mRNA IRESs. Cold Spring Harb. Perspect. Biol 5, a011569. doi:10.1101/cshperspect.a011569 23378589 PMC3552511

[R124] Janin M, Ortiz-Barahona V, de Moura MC, Martinez-Cardus A, Llinas-Arias P, Soler M, (2019). Epigenetic loss of RNA-methyltransferase NSUN5 in glioma targets ribosomes to drive a stress adaptive translational program. Acta Neuropathol. 138, 1053–1074. doi:10.1007/s00401-019-02062-4 31428936 PMC6851045

[R125] Jiang J, Aduri R, Chow CS, and SantaLucia J Jr, (2014). Structure modulation of helix 69 from *Escherichia coli* 23S ribosomal RNA by pseudouridylations. Nucleic Acids Res. 42, 3971–3981. doi:10.1093/nar/gkt1329 24371282 PMC3973299

[R126] Johnson LK, Johnson RW, and Strehler BL (1975). Cardiac hypertrophy, aging and changes in cardiac ribosomal RNA gene dosage in man. J. Mol. Cell. Cardiol 7, 125–133. doi:10.1016/0022-2828(75)90014-0 123595

[R127] Johnson R, and Strehler BL (1972). Loss of genes coding for ribosomal RNA in ageing brain cells. Nature 240, 412–414. doi:10.1038/240412a0 4564320

[R128] Jones KL, Smith DW, Harvey MA, Hall BD, and Quan L (1975). Older paternal age and fresh gene mutation: data on additional disorders. J. Pediatr 86, 84–88. doi:10.1016/s0022-3476(75)80709-8 1110452

[R129] Jones NC, Lynn ML, Gaudenz K, Sakai D, Aoto K, Rey JP, (2008). Prevention of the neurocristopathy Treacher Collins syndrome through inhibition of p53 function. Nat. Med 14, 125–133. doi:10.1038/nm1725 18246078 PMC3093709

[R130] Karwan R, Bennett JL, and Clayton DA (1991). Nuclear RNase MRP processes RNA at multiple discrete sites: interaction with an upstream G box is required for subsequent downstream cleavages. Genes Dev. 5, 1264–1276. doi:10.1101/gad.5.7.1264 2065976

[R131] Kass S, Craig N, and Sollner-Webb B (1987). Primary processing of mammalian rRNA involves two adjacent cleavages and is not species specific. Mol. Cell. Biol 7, 2891–2898. doi:10.1128/mcb.7.8.2891 3670298 PMC367908

[R132] Kass S, Tyc K, Steitz JA, and Sollner-Webb B (1990). The U3 small nucleolar ribonucleoprotein functions in the first step of preribosomal RNA processing. Cell 60, 897–908. doi:10.1016/0092-8674(90)90338-f 2156625

[R133] Kempers-Veenstra AE, Oliemans J, Offenberg H, Dekker AF, Piper PW, Planta RJ, (1986). 3’-End formation of transcripts from the yeast rRNA operon. EMBO J. 5, 2703–2710. doi:10.1002/j.1460-2075.1986.tb04554.x 3780675 PMC1167172

[R134] Kennedy S, Wang D, and Ruvkun G (2004). A conserved siRNA-degrading RNase negatively regulates RNA interference in *C. elegans*. Nature 427, 645–649. doi:10.1038/nature02302 14961122

[R135] Khalid F, Phan T, Qiang M, Maity P, Lasser T, Wiese S, (2023). TFIIH mutations can impact on translational fidelity of the ribosome. Hum. Mol. Genet 32, 1102–1113. doi:10.1093/hmg/ddac268 36308430 PMC10026254

[R136] Khatter H, Myasnikov AG, Natchiar SK, and Klaholz BP (2015). Structure of the human 80S ribosome. Nature 520, 640–645. doi:10.1038/nature14427 25901680

[R137] Khoshnevis S, Dreggors-Walker RE, Marchand V, Motorin Y, and Ghalei H (2022). Ribosomal RNA 2’-O-methylations regulate translation by impacting ribosome dynamics. Proc. Natl. Acad. Sci. U. S. A 119, e2117334119. doi:10.1073/pnas.2117334119 35294285 PMC8944910

[R138] Kim JH, Dilthey AT, Nagaraja R, Lee HS, Koren S, Dudekula D, (2018). Variation in human chromosome 21 ribosomal RNA genes characterized by TAR cloning and long-read sequencing. Nucleic Acids Res. 46, 6712–6725. doi:10.1093/nar/gky442 29788454 PMC6061828

[R139] Kim S, Li Q, Dang CV, and Lee LA (2000). Induction of ribosomal genes and hepatocyte hypertrophy by adenovirus-mediated expression of c-Myc *in vivo*. Proc. Natl. Acad. Sci. U. S. A 97, 11198–11202. doi:10.1073/pnas.200372597 11005843 PMC17177

[R140] Kiss T, Marshallsay C, and Filipowicz W (1992). 7–2/MRP RNAs in plant and mammalian cells: association with higher order structures in the nucleolus. EMBO J. 11, 3737–3746. doi:10.1002/j.1460-2075.1992.tb05459.x 1382978 PMC556834

[R141] Knight SW, Heiss NS, Vulliamy TJ, Greschner S, Stavrides G, Pai GS, (1999). X-linked dyskeratosis congenita is predominantly caused by missense mutations in the DKC1 gene. Am. J. Hum. Genet 65, 50–58. doi:10.1086/302446 10364516 PMC1378074

[R142] Knorr AG, Schmidt C, Tesina P, Berninghausen O, Becker T, Beatrix B, (2019). Ribosome-NatA architecture reveals that rRNA expansion segments coordinate N-terminal acetylation. Nat. Struct. Mol. Biol 26, 35–39. doi:10.1038/s41594-018-0165-y 30559462

[R143] Kobayashi T (2011). Regulation of ribosomal RNA gene copy number and its role in modulating genome integrity and evolutionary adaptability in yeast. Cell Mol. Life Sci 68, 1395–1403. doi:10.1007/s00018-010-0613-2 21207101 PMC3064901

[R144] Koch S, Garcia Gonzalez O, Assfalg R, Schelling A, Schafer P, Scharffetter-Kochanek K, (2014). Cockayne syndrome protein A is a transcription factor of RNA polymerase I and stimulates ribosomal biogenesis and growth. Cell Cycle 13, 2029–2037. doi:10.4161/cc.29018 24781187 PMC4111694

[R145] Kossinova O, Malygin A, Krol A, and Karpova G (2014). The SBP2 protein central to selenoprotein synthesis contacts the human ribosome at expansion segment 7L of the 28S rRNA. RNA 20, 1046–1056. doi:10.1261/rna.044917.114 24850884 PMC4114684

[R146] Krauer N, Rauscher R, and Polacek N (2021). tRNA synthetases are recruited to yeast ribosomes by rRNA expansion segment 7L but do not require association for functionality. Noncoding RNA 7, 73. doi:10.3390/ncrna7040073 34842814 PMC8628890

[R147] Krishnan P, Ghosh S, Wang B, Heyns M, Graham K, Mackey JR, (2016). Profiling of small nucleolar RNAs by next generation sequencing: potential new players for breast cancer prognosis. PLoS One 11, e0162622. doi:10.1371/journal.pone.0162622 27631501 PMC5025248

[R148] Krogh N, Jansson MD, Hafner SJ, Tehler D, Birkedal U, Christensen-Dalsgaard M, (2016). Profiling of 2’-O-Me in human rRNA reveals a subset of fractionally modified positions and provides evidence for ribosome heterogeneity. Nucleic Acids Res. 44, 7884–7895. doi:10.1093/nar/gkw482 27257078 PMC5027482

[R149] Kufel J, Dichtl B, and Tollervey D (1999). Yeast Rnt1p is required for cleavage of the pre-ribosomal RNA in the 3’ ETS but not the 5’ ETS. RNA 5, 909–917. doi:10.1017/s135583829999026x 10411134 PMC1369815

[R150] Lafontaine DLJ, Riback JA, Bascetin R, and Brangwynne CP (2021). The nucleolus as a multiphase liquid condensate. Nat. Rev. Mol. Cell Biol 22, 165–182. doi:10.1038/s41580-020-0272-6 32873929

[R151] Lamont RE, Loredo-Osti J, Roslin NM, Mauthe J, Coghlan G, Nylen E, (2005). A locus for Bowen-Conradi syndrome maps to chromosome region 12p13.3. Am. J. Med. Genet. Part A 132A, 136–143. doi:10.1002/ajmg.a.30420 15578624

[R152] Lancaster L, Lambert NJ, Maklan EJ, Horan LH, and Noller HF (2008). The sarcin-ricin loop of 23S rRNA is essential for assembly of the functional core of the 50S ribosomal subunit. RNA 14, 1999–2012. doi:10.1261/rna.1202108 18755834 PMC2553751

[R153] Lang K, Erlacher M, Wilson DN, Micura R, and Polacek N (2008). The role of 23S ribosomal RNA residue A2451 in peptide bond synthesis revealed by atomic mutagenesis. Chem. Biol 15, 485–492. doi:10.1016/j.chembiol.2008.03.014 18439847

[R154] Langhendries JL, Nicolas E, Doumont G, Goldman S, and Lafontaine DL (2016). The human box C/D snoRNAs U3 and U8 are required for pre-rRNA processing and tumorigenesis. Oncotarget 7, 59519–59534. doi:10.18632/oncotarget.11148 27517747 PMC5312328

[R155] Laugel V, Dalloz C, Stary A, Cormier-Daire V, Desguerre I, Renouil M, (2008). Deletion of 5’ sequences of the CSB gene provides insight into the pathophysiology of Cockayne syndrome. Eur. J. Hum. Genet 16, 320–327. doi:10.1038/sj.ejhg.5201991 18183039

[R156] Learned RM, Cordes S, and Tjian R (1985). Purification and characterization of a transcription factor that confers promoter specificity to human RNA polymerase I. Mol. Cell. Biol 5, 1358–1369. doi:10.1128/mcb.5.6.1358 3929071 PMC366865

[R157] Learned RM, Learned TK, Haltiner MM, and Tjian RT (1986). Human rRNA transcription is modulated by the coordinate binding of two factors to an upstream control element. Cell 45, 847–857. doi:10.1016/0092-8674(86)90559-3 3708692

[R158] Learned RM, Smale ST, Haltiner MM, and Tjian R (1983). Regulation of human ribosomal RNA transcription. Proc. Natl. Acad. Sci. U. S. A 80, 3558–3562. doi:10.1073/pnas.80.12.3558 6304717 PMC394088

[R159] Lebaron S, Schneider C, van Nues RW, Swiatkowska A, Walsh D, Bottcher B, (2012). Proofreading of pre-40S ribosome maturation by a translation initiation factor and 60S subunits. Nat. Struct. Mol. Biol 19, 744–753. doi:10.1038/nsmb.2308 22751017 PMC3654374

[R160] Lebedev A, Scharffetter-Kochanek K, and Iben S (2008). Truncated Cockayne syndrome B protein represses elongation by RNA polymerase I. J. Mol. Biol 382, 266–274. doi:10.1016/j.jmb.2008.07.018 18656484

[R161] Lehmann AR, Thompson AF, Harcourt SA, Stefanini M, and Norris PG (1993). Cockayne’s syndrome: correlation of clinical features with cellular sensitivity of RNA synthesis to UV irradiation. J. Med. Genet 30, 679–682. doi:10.1136/jmg.30.8.679 7692050 PMC1016498

[R162] Leppek K, Fujii K, Quade N, Susanto TT, Boehringer D, Lenarčič T, (2020). Gene- and species-specific Hox mRNA translation by ribosome expansion segments. Mol. Cell 80, 980–995.e13. doi:10.1016/j.molcel.2020.10.023 33202249 PMC7769145

[R163] Liang XH, Liu Q, and Fournier MJ (2007). rRNA modifications in an intersubunit bridge of the ribosome strongly affect both ribosome biogenesis and activity. Mol. Cell 28, 965–977. doi:10.1016/j.molcel.2007.10.012 18158895

[R164] Liang XH, Liu Q, and Fournier MJ (2009). Loss of rRNA modifications in the decoding center of the ribosome impairs translation and strongly delays pre-rRNA processing. RNA 15, 1716–1728. doi:10.1261/rna.1724409 19628622 PMC2743053

[R165] Liano D, Chowdhury S, and Di Antonio M (2021). Cockayne syndrome B protein selectively resolves and interact with intermolecular DNA G-quadruplex structures. J. Am. Chem. Soc 143, 20988–21002. doi:10.1021/jacs.1c10745 34855372

[R166] Liebhaber SA, Wolf S, and Schlessinger D (1978). Differences in rRNA metabolism of primary and SV40-transformed human fibroblasts. Cell 13, 121–127. doi:10.1016/0092-8674(78)90143-5 202397

[R167] Lindahl L, Archer RH, and Zengel JM (1992). A new rRNA processing mutant of *Saccharomyces cerevisiae*. Nucleic Acids Res. 20, 295–301. doi:10.1093/nar/20.2.295 1741255 PMC310369

[R168] Lomakin IB, and Steitz TA (2013). The initiation of mammalian protein synthesis and mRNA scanning mechanism. Nature 500, 307–311. doi:10.1038/nature12355 23873042 PMC3748252

[R169] López MD, Rosenblad MA, and Samuelsson T (2009). Conserved and variable domains of RNase MRP RNA. RNA Biol. 6, 208–221. doi:10.4161/rna.6.3.8584 19395864

[R170] Lygerou Z, Allmang C, Tollervey D, and Seraphin B (1996). Accurate processing of a eukaryotic precursor ribosomal RNA by ribonuclease MRP *in vitro*. Science 272, 268–270. doi:10.1126/science.272.5259.268 8602511

[R171] Lygerou Z, Mitchell P, Petfalski E, Seraphin B, and Tollervey D (1994). The POP1 gene encodes a protein component common to the RNase MRP and RNase P ribonucleoproteins. Genes Dev. 8, 1423–1433. doi:10.1101/gad.8.12.1423 7926742

[R172] Maccarty WC (1936). The value of the macronucleolus in the cancer problem. Am. J. Cancer 26, 529–532. doi:10.1158/ajc.1936.529

[R173] Maden BE, Vaughan MH, Warner JR, and Darnell JE (1969). Effects of valine deprivation on ribosome formation in HeLa cells. J. Mol. Biol 45, 265–275. doi:10.1016/0022-2836(69)90104-1 5367028

[R174] Mahajan PB, and Thompson EA (1990). Hormonal regulation of transcription of rDNA. Purification and characterization of the hormone-regulated transcription factor IC. J. Biol. Chem 265, 16225–16233. doi:10.1016/s0021-9258(17)46212-0 2398050

[R175] Maizels N (1976). Dictyostelium 17S, 25S, and 5S rDNAs lie within a 38,000 base pair repeated unit. Cell 9, 431–438. doi:10.1016/0092-8674(76)90088-x 1033038

[R176] Malinovskaya EM, Ershova ES, Golimbet VE, Porokhovnik LN, Lyapunova NA, Kutsev SI, (2018). Copy number of human ribosomal genes with aging: unchanged mean, but narrowed range and decreased variance in elderly group. Front. Genet 9, 306. doi:10.3389/fgene.2018.00306 30131826 PMC6090032

[R177] Mao J, Appel B, Schaack J, Sharp S, Yamada H, and Soll D (1982). The 5S RNA genes of *Schizosaccharomyces pombe*. Nucleic Acids Res. 10, 487–500. doi:10.1093/nar/10.2.487 6278416 PMC326152

[R178] Marcel V, Ghayad SE, Belin S, Therizols G, Morel AP, Solano-Gonzalez E, (2013). p53 acts as a safeguard of translational control by regulating fibrillarin and rRNA methylation in cancer. Cancer Cell 24, 318–330. doi:10.1016/j.ccr.2013.08.013 24029231 PMC7106277

[R179] Marcel V, Kielbassa J, Marchand V, Natchiar KS, Paraqindes H, Nguyen Van Long F, (2020). Ribosomal RNA 2’O-methylation as a novel layer of inter-tumour heterogeneity in breast cancer. Nar. Cancer 2, zcaa036. doi:10.1093/narcan/zcaa036 34316693 PMC8210124

[R180] Mars JC, Tremblay MG, Valere M, Sibai DS, Sabourin-Felix M, Lessard F, (2020). The chemotherapeutic agent CX-5461 irreversibly blocks RNA polymerase I initiation and promoter release to cause nucleolar disruption, DNA damage and cell inviability. Nar. Cancer 2, zcaa032. doi:10.1093/narcan/zcaa032 33196044 PMC7646227

[R181] Mattioli F, Worpenberg L, Li CT, Ibrahim N, Naz S, Sharif S, (2023). Biallelic variants in NSUN6 cause an autosomal recessive neurodevelopmental disorder. Genet. Med 25, 100900. doi:10.1016/j.gim.2023.100900 37226891

[R182] Mayer C, Bierhoff H, and Grummt I (2005). The nucleolus as a stress sensor: JNK2 inactivates the transcription factor TIF-IA and down-regulates rRNA synthesis. Genes Dev. 19, 933–941. doi:10.1101/gad.333205 15805466 PMC1080132

[R183] Mayer C, Zhao J, Yuan X, and Grummt I (2004). mTOR-dependent activation of the transcription factor TIF-IA links rRNA synthesis to nutrient availability. Genes Dev. 18, 423–434. doi:10.1101/gad.285504 15004009 PMC359396

[R184] Mayne LV, and Lehmann AR (1982). Failure of RNA synthesis to recover after UV irradiation: an early defect in cells from individuals with Cockayne’s syndrome and xeroderma pigmentosum. Cancer Res. 42, 1473–1478. doi:10.1016/0027-5107(82)90047-1 6174225

[R185] McKusick VA, Eldridge R, Hostetler JA, Ruangwit U, and Egeland JA (1965). Dwarfism in the amish. Ii. Cartilage-hair hypoplasia. Bull. Johns Hopkins Hosp 116, 285–326. 14284412

[R186] Medvedev ZA (1972). Repetition of molecular-genetic information as a possible factor in evolutionary changes of life span. Exp. Gerontol 7, 227–238. doi:10.1016/0531-5565(72)90012-5 5073315

[R187] Meier UT, and Blobel G (1994). NAP57, a mammalian nucleolar protein with a putative homolog in yeast and bacteria. J. Cell Biol 127, 1505–1514. doi:10.1083/jcb.127.6.1505 7798307 PMC2120319

[R188] Mestre-Fos S, Penev PI, Suttapitugsakul S, Hu M, Ito C, Petrov AS, (2019). G-quadruplexes in human ribosomal RNA. J. Mol. Biol 431, 1940–1955. doi:10.1016/j.jmb.2019.03.010 30885721 PMC8064279

[R189] Meyer B, Wurm JP, Kotter P, Leisegang MS, Schilling V, Buchhaupt M, (2011). The Bowen–Conradi syndrome protein Nep1 (Emg1) has a dual role in eukaryotic ribosome biogenesis, as an essential assembly factor and in the methylation of Ψ1191 in yeast 18S rRNA. Nucleic Acids Res. 39, 1526–1537. doi:10.1093/nar/gkq931 20972225 PMC3045603

[R190] Meyer B, Wurm JP, Sharma S, Immer C, Pogoryelov D, Kotter P, (2016). Ribosome biogenesis factor Tsr3 is the aminocarboxypropyl transferase responsible for 18S rRNA hypermodification in yeast and humans. Nucleic Acids Res. 44, 4304–4316. doi:10.1093/nar/gkw244 27084949 PMC4872110

[R191] Milkereit P, and Tschochner H (1998). A specialized form of RNA polymerase I, essential for initiation and growth-dependent regulation of rRNA synthesis, is disrupted during transcription. EMBO J. 17, 3692–3703. doi:10.1093/emboj/17.13.3692 9649439 PMC1170705

[R192] Mitchell JR, Wood E, and Collins K (1999). A telomerase component is defective in the human disease dyskeratosis congenita. Nature 402, 551–555. doi:10.1038/990141 10591218

[R193] Mitchell P, Petfalski E, Shevchenko A, Mann M, and Tollervey D (1997). The exosome: a conserved eukaryotic RNA processing complex containing multiple 3’-->5’ exoribonucleases. Cell 91, 457–466. doi:10.1016/s0092-8674(00)80432-8 9390555

[R194] Moazed D, and Noller HF (1986). Transfer RNA shields specific nucleotides in 16S ribosomal RNA from attack by chemical probes. Cell 47, 985–994. doi:10.1016/0092-8674(86)90813-5 2430725

[R195] Moazed D, and Noller HF (1987). Chloramphenicol, erythromycin, carbomycin and vernamycin B protect overlapping sites in the peptidyl transferase region of 23S ribosomal RNA. Biochimie 69, 879–884. doi:10.1016/0300-9084(87)90215-x 3122849

[R196] Moazed D, and Noller HF (1990). Binding of tRNA to the ribosomal A and P sites protects two distinct sets of nucleotides in 16 S rRNA. J. Mol. Biol 211, 135–145. doi:10.1016/0022-2836(90)90016-f 2405162

[R197] Montanaro L, Brigotti M, Clohessy J, Barbieri S, Ceccarelli C, Santini D, (2006). Dyskerin expression influences the level of ribosomal RNA pseudo-uridylation and telomerase RNA component in human breast cancer. J. Pathology 210, 10–18. doi:10.1002/path.2023 16841302

[R198] Moorefield B, Greene EA, and Reeder RH (2000). RNA polymerase I transcription factor Rrn3 is functionally conserved between yeast and human. Proc. Natl. Acad. Sci. U. S. A 97, 4724–4729. doi:10.1073/pnas.080063997 10758157 PMC18300

[R199] Murayama A, Ohmori K, Fujimura A, Minami H, Yasuzawa-Tanaka K, Kuroda T, (2008). Epigenetic control of rDNA loci in response to intracellular energy status. Cell 133, 627–639. doi:10.1016/j.cell.2008.03.030 18485871

[R200] Nachmani D, Bothmer AH, Grisendi S, Mele A, Bothmer D, Lee JD, (2019). Germline NPM1 mutations lead to altered rRNA 2’-O-methylation and cause dyskeratosis congenita. Nat. Genet 51, 1518–1529. doi:10.1038/s41588-019-0502-z 31570891 PMC6858547

[R201] Nance MA, and Berry SA (1992). Cockayne syndrome: review of 140 cases. Am. J. Med. Genet 42, 68–84. doi:10.1002/ajmg.1320420115 1308368

[R202] Narayanan DL, Shukla A, Kausthubham N, Bhavani GS, Shah H, Mortier G, (2019). An emerging ribosomopathy affecting the skeleton due to biallelic variations in NEPRO. Am. J. Med. Genet. Part A 179, 1709–1717. doi:10.1002/ajmg.a.61267 31250547

[R203] Nierhaus KH, and Dohme F (1974). Total reconstitution of functionally active 50S ribosomal subunits from *Escherichia coli*. Proc. Natl. Acad. Sci. U. S. A 71, 4713–4717. doi:10.1073/pnas.71.12.4713 4612527 PMC433966

[R204] Nissen P, Hansen J, Ban N, Moore PB, and Steitz TA (2000). The structural basis of ribosome activity in peptide bond synthesis. Science 289, 920–930. doi:10.1126/science.289.5481.920 10937990

[R205] Noller HF, and Chaires JB (1972). Functional modification of 16S ribosomal RNA by kethoxal. Proc. Natl. Acad. Sci. U. S. A 69, 3115–3118. doi:10.1073/pnas.69.11.3115 4564202 PMC389716

[R206] Noller HF, Hoffarth V, and Zimniak L (1992). Unusual resistance of peptidyl transferase to protein extraction procedures. Science 256, 1416–1419. doi:10.1126/science.1604315 1604315

[R207] Noller HF, Kop J, Wheaton V, Brosius J, Gutell RR, Kopylov AM, (1981). Secondary structure model for 23S ribosomal RNA. Nucleic Acids Res. 9, 6167–6189. doi:10.1093/nar/9.22.6167 7031608 PMC327592

[R208] Nurk S, Koren S, Rhie A, Rautiainen M, Bzikadze AV, Mikheenko A, (2022). The complete sequence of a human genome. Science 376, 44–53. doi:10.1126/science.abj6987 35357919 PMC9186530

[R209] Ogle JM, Brodersen DE, Clemons WM Jr., Tarry MJ, Carter AP, and Ramakrishnan V (2001). Recognition of cognate transfer RNA by the 30S ribosomal subunit. Science 292, 897–902. doi:10.1126/science.1060612 11340196

[R210] Ogle JM, Murphy FV, Tarry MJ, and Ramakrishnan V (2002). Selection of tRNA by the ribosome requires a transition from an open to a closed form. Cell 111, 721–732. doi:10.1016/s0092-8674(02)01086-3 12464183

[R211] Ojha S, Malla S, and Lyons SM (2020). snoRNPs: functions in ribosome biogenesis. Biomolecules 10, 783. doi:10.3390/biom10050783 32443616 PMC7277114

[R212] Okur MN, Lee JH, Osmani W, Kimura R, Demarest TG, Croteau DL, (2020). Cockayne syndrome group A and B proteins function in rRNA transcription through nucleolin regulation. Nucleic Acids Res. 48, 2473–2485. doi:10.1093/nar/gkz1242 31970402 PMC7049711

[R213] Oshima J, Sidorova JM, and Monnat RJW (2017). Werner syndrome: clinical features, pathogenesis and potential therapeutic interventions. Ageing Res. Rev 33, 105–114. doi:10.1016/j.arr.2016.03.002 26993153 PMC5025328

[R214] Paramasivam A, Meena AK, Venkatapathi C, Pitceathly RDS, and Thangaraj K (2020). Novel biallelic NSUN3 variants cause early-onset mitochondrial encephalomyopathy and seizures. J. Mol. Neurosci 70, 1962–1965. doi:10.1007/s12031-020-01595-8 32488845 PMC7658056

[R215] Parker KA, and Steitz JA (1987). Structural analysis of the human U3 ribonucleoprotein particle reveal a conserved sequence available for base pairing with pre-rRNA. Mol. Cell. Biol 7, 2899–2913. doi:10.1128/mcb.7.8.2899-2913.1987 2959855 PMC367909

[R216] Peculis BA, and Steitz JA (1993). Disruption of U8 nucleolar snRNA inhibits 5.8S and 28S rRNA processing in the Xenopus oocyte. Cell 73, 1233–1245. doi:10.1016/0092-8674(93)90651-6 8513505

[R217] Penzo M, Rocchi L, Brugiere S, Carnicelli D, Onofrillo C, Coute Y, (2015). Human ribosomes from cells with reduced dyskerin levels are intrinsically altered in translation. FASEB J. 29, 3472–3482. doi:10.1096/fj.15-270991 25934701

[R218] PerezGrovas-Saltijeral A, Rajkumar AP, and Knight HM (2023). Differential expression of m(5)C RNA methyltransferase genes NSUN6 and NSUN7 in Alzheimer’s disease and traumatic brain injury. Mol. Neurobiol 60, 2223–2235. doi:10.1007/s12035-022-03195-6 36646969 PMC9984329

[R219] Petes TD, Hereford LM, and Skryabin KG (1978). Characterization of two types of yeast ribosomal DNA genes. J. Bacteriol 134, 295–305. doi:10.1128/jb.134.1.295-305.1978 348684 PMC222246

[R220] Peyroche G, Milkereit P, Bischler N, Tschochner H, Schultz P, Sentenac A, (2000). The recruitment of RNA polymerase I on rDNA is mediated by the interaction of the A43 subunit with Rrn3. EMBO J. 19, 5473–5482. doi:10.1093/emboj/19.20.5473 11032814 PMC314014

[R221] Philipson L (1961). Chromatographic separation, and characteristics of nucleic acids from HeLa cells. J. General Physiology 44, 899–910. doi:10.1085/jgp.44.5.899 PMC2195130 13735276

[R222] Pietrzak M, Rempala G, Nelson PT, Zheng JJ, and Hetman M (2011). Epigenetic silencing of nucleolar rRNA genes in Alzheimer’s disease. PLoS One 6, e22585. doi:10.1371/journal.pone.0022585 21799908 PMC3142181

[R223] Pillon MC, Hsu AL, Krahn JM, Williams JG, Goslen KH, Sobhany M, (2019). Cryo-EM reveals active site coordination within a multienzyme pre-rRNA processing complex. Nat. Struct. Mol. Biol 26, 830–839. doi:10.1038/s41594-019-0289-8 31488907 PMC6733591

[R224] Pillon MC, Sobhany M, Borgnia MJ, Williams JG, and Stanley RE (2017). Grc3 programs the essential endoribonuclease Las1 for specific RNA cleavage. Proc. Natl. Acad. Sci. U. S. A 114, E5530–E5538. doi:10.1073/pnas.1703133114 28652339 PMC5514736

[R225] Pillon MC, Sobhany M, and Stanley RE (2018). Characterization of the molecular crosstalk within the essential Grc3/Las1 pre-rRNA processing complex. RNA 24, 721–738. doi:10.1261/rna.065037.117 29440475 PMC5900568

[R226] Piper PW, Bellatin JA, and Lockheart A (1983). Altered maturation of sequences at the 3’ terminus of 5S gene transcripts in a *Saccharomyces cerevisiae* mutant that lacks a RNA processing endonuclease. EMBO J. 2, 353–359. doi:10.1002/j.1460-2075.1983.tb01430.x 11894949 PMC555140

[R227] Pober BR (2010). Williams–beuren syndrome. N. Engl. J. Med 362, 239–252. doi:10.1056/nejmra0903074 20089974

[R228] Polikanov YS, Steitz TA, and Innis CA (2014). A proton wire to couple aminoacyl-tRNA accommodation and peptide-bond formation on the ribosome. Nat. Struct. Mol. Biol 21, 787–793. doi:10.1038/nsmb.2871 25132179 PMC4156881

[R229] Popov A, Smirnov E, Kováčik L, Raška O, Hagen G, Stixova L, (2013). Duration of the first steps of the human rRNA processing. Nucleus 4, 134–141. doi:10.4161/nucl.23985 23412654 PMC3621745

[R230] Prestayko AW, Tonato M, and Busch H (1970). Low molecular weight RNA associated with 28 s nucleolar RNA. J. Mol. Biol 47, 505–515. doi:10.1016/0022-2836(70)90318-9 5418169

[R231] Prieto JL, and McStay B (2007). Recruitment of factors linking transcription and processing of pre-rRNA to NOR chromatin is UBF-dependent and occurs independent of transcription in human cells. Genes Dev. 21, 2041–2054. doi:10.1101/gad.436707 17699751 PMC1948859

[R232] Reimer G, Scheer U, Peters JM, and Tan EM (1986). Immunolocalization and partial characterization of a nucleolar autoantigen (PM-Scl) associated with polymyositis/scleroderma overlap syndromes. J. Immunol 137, 3802–3808. doi:10.4049/jimmunol.137.12.3802 3537125

[R233] Remmelzwaal PC, Verhagen MV, Jongbloed JDH, van den Akker PC, Veenstra-Knol HE, and Hitzert MM (2023). Expanding the phenotype of anauxetic dysplasia caused by biallelic NEPRO mutations: a case report. Am. J. Med. Genet. Part A 191, 2440–2445. doi:10.1002/ajmg.a.63316 37294112

[R234] Ren R, Deng L, Xue Y, Suzuki K, Zhang W, Yu Y, (2017). Visualization of aging-associated chromatin alterations with an engineered TALE system. Cell Res. 27, 483–504. doi:10.1038/cr.2017.18 28139645 PMC5385610

[R235] Ridanpaa M, van Eenennaam H, Pelin K, Chadwick R, Johnson C, Yuan B, (2001). Mutations in the RNA component of RNase MRP cause a pleiotropic human disease, cartilage-hair hypoplasia. Cell 104, 195–203. doi:10.1016/s0092-8674(01)00205-7 11207361

[R236] Rinke J, and Steitz JA (1982). Precursor molecules of both human 5S ribosomal RNA and transfer RNAs are bound by a cellular protein reactive with anti-La lupus antibodies. Cell 29, 149–159. doi:10.1016/0092-8674(82)90099-x 7105180

[R237] Ritossa FM, Atwood KC, and Spiegelman S (1966). A molecular explanation of the bobbed mutants of Drosophila as partial deficiencies of “ribosomal” DNA. Genetics 54, 819–834. doi:10.1093/genetics/54.3.819 5970623 PMC1211204

[R238] Robertson N, Shchepachev V, Wright D, Turowski TW, Spanos C, Helwak A, (2022). A disease-linked lncRNA mutation in RNase MRP inhibits ribosome synthesis. Nat. Commun 13, 649. doi:10.1038/s41467-022-28295-8 35115551 PMC8814244

[R239] Rong B, Zhang Q, Wan J, Xing S, Dai R, Li Y, (2020). Ribosome 18S m(6) A methyltransferase METTL5 promotes translation initiation and breast cancer cell growth. Cell Rep. 33, 108544. doi:10.1016/j.celrep.2020.108544 33357433

[R240] Rouquette J, Choesmel V, and Gleizes PE (2005). Nuclear export and cytoplasmic processing of precursors to the 40S ribosomal subunits in mammalian cells. EMBO J. 24, 2862–2872. doi:10.1038/sj.emboj.7600752 16037817 PMC1187937

[R241] Ruggero D, Grisendi S, Piazza F, Rego E, Mari F, Rao PH, (2003). Dyskeratosis congenita and cancer in mice deficient in ribosomal RNA modification. Science 299, 259–262. doi:10.1126/science.1079447 12522253

[R242] Sadian Y, Baudin F, Tafur L, Murciano B, Wetzel R, Weis F, (2019). Molecular insight into RNA polymerase I promoter recognition and promoter melting. Nat. Commun 10, 5543. doi:10.1038/s41467-019-13510-w 31804486 PMC6895186

[R243] Satoh A, Brace CS, Rensing N, Cliften P, Wozniak DF, Herzog ED, (2013). Sirt1 extends life span and delays aging in mice through the regulation of Nk2 homeobox 1 in the DMH and LH. Cell Metab. 18, 416–430. doi:10.1016/j.cmet.2013.07.013 24011076 PMC3794712

[R244] Savage SA, and Niewisch MR (1993). “Dyskeratosis congenita and related telomere biology disorders,” in GeneReviews((R)). Editors Adam MP, Feldman J, Mirzaa GM, Pagon RA, Wallace SE, Bean LJH, (Seattle (WA): National Library of Medicine).

[R245] Scheibye-Knudsen M, Tseng A, Borch Jensen M, Scheibye-Alsing K, Fang EF, Iyama T, (2016). Cockayne syndrome group A and B proteins converge on transcription-linked resolution of non-B DNA. Proc. Natl. Acad. Sci. U. S. A 113, 12502–12507. doi:10.1073/pnas.1610198113 27791127 PMC5098674

[R246] Scherrer K, and Darnell JE (1962). Sedimentation characteristics of rapidly labelled RNA from HeLa cells. Biochem. Biophysical Res. Commun 7, 486–490. doi:10.1016/0006-291x(62)90341-8 14498283

[R247] Scherrer K, Latham H, and Darnell JE (1963). Demonstration of an unstable RNA and of a precursor to ribosomal RNA in HeLa cells. Proc. Natl. Acad. Sci. U. S. A 49, 240–248. doi:10.1073/pnas.49.2.240 13991616 PMC299789

[R248] Schmickel RD, Chu EH, Trosko JE, and Chang CC (1977). Cockayne syndrome: a cellular sensitivity to ultraviolet light. Pediatrics 60, 135–139. doi:10.1542/peds.60.2.135 887325

[R249] Schmitt ME, and Clayton DA (1992). Yeast site-specific ribonucleoprotein endoribonuclease MRP contains an RNA component homologous to mammalian RNase MRP RNA and essential for cell viability. Genes Dev. 6, 1975–1985. doi:10.1101/gad.6.10.1975 1398074

[R250] Schmitt ME, and Clayton DA (1993). Nuclear RNase MRP is required for correct processing of pre-5.8S rRNA in *Saccharomyces cerevisiae*. Mol. Cell Biol 13, 7935–7941. doi:10.1128/mcb.13.12.7935 8247008 PMC364865

[R251] Schosserer M, Minois N, Angerer TB, Amring M, Dellago H, Harreither E, (2015). Methylation of ribosomal RNA by NSUN5 is a conserved mechanism modulating organismal lifespan. Nat. Commun 6, 6158. doi:10.1038/ncomms7158 25635753 PMC4317494

[R252] Schuller JM, Falk S, Fromm L, Hurt E, and Conti E (2018). Structure of the nuclear exosome captured on a maturing preribosome. Science 360, 219–222. doi:10.1126/science.aar5428 29519915

[R253] Schulze H, and Nierhaus KH (1982). Minimal set of ribosomal components for reconstitution of the peptidyltransferase activity. EMBO J. 1, 609–613. doi:10.1002/j.1460-2075.1982.tb01216.x 6765232 PMC553095

[R254] Selker EU, Yanofsky C, Driftmier K, Metzenberg RL, Alzner-DeWeerd B, and RajBhandary UL (1981). Dispersed 5S RNA genes in N. crassa: structure, expression and evolution. Cell 24, 819–828. doi:10.1016/0092-8674(81)90107-0 6454495

[R255] Selmer M, Dunham CM, Murphy F. V. t., Weixlbaumer A, Petry S, Kelley AC, (2006). Structure of the 70S ribosome complexed with mRNA and tRNA. Science 313, 1935–1942. doi:10.1126/science.1131127 16959973

[R256] Sepich-Poore C, Zheng Z, Schmitt E, Wen K, Zhang ZS, Cui XL, (2022). The METTL5-TRMT112 N(6)-methyladenosine methyltransferase complex regulates mRNA translation via 18S rRNA methylation. J. Biol. Chem 298, 101590. doi:10.1016/j.jbc.2022.101590 35033535 PMC8857481

[R257] Shankar V, Rauscher R, Reuther J, Gharib WH, Koch M, and Polacek N (2020). rRNA expansion segment 27Lb modulates the factor recruitment capacity of the yeast ribosome and shapes the proteome. Nucleic Acids Res. 48, 3244–3256. doi:10.1093/nar/gkaa003 31960048 PMC7102955

[R258] Shao S, Murray J, Brown A, Taunton J, Ramakrishnan V, and Hegde RS (2016). Decoding mammalian ribosome-mRNA states by translational GTPase complexes. Cell 167, 1229–1240.e15. doi:10.1016/j.cell.2016.10.046 27863242 PMC5119991

[R259] Sharma S, Langhendries JL, Watzinger P, Kotter P, Entian KD, and Lafontaine DL (2015). Yeast Kre33 and human NAT10 are conserved 18S rRNA cytosine acetyltransferases that modify tRNAs assisted by the adaptor Tan1/THUMPD1. Nucleic Acids Res. 43, 2242–2258. doi:10.1093/nar/gkv075 25653167 PMC4344512

[R260] Sharma S, Yang J, Watzinger P, Kotter P, and Entian KD (2013). Yeast Nop2 and Rcm1 methylate C2870 and C2278 of the 25S rRNA, respectively. Nucleic Acids Res. 41, 9062–9076. doi:10.1093/nar/gkt679 23913415 PMC3799443

[R261] Sharp S, Garcia A, Cooley L, and Soll D (1984). Transcriptionally active and inactive gene repeats within the*D. meianogaster*5S RNA gene cluster. Nucleic Acids Res. 12, 7617–7632. doi:10.1093/nar/12.20.7617 6093044 PMC320189

[R262] Shiue CN, Berkson RG, and Wright AP (2009). c-Myc induces changes in higher order rDNA structure on stimulation of quiescent cells. Oncogene 28, 1833–1842. doi:10.1038/onc.2009.21 19270725

[R263] Shobuike T, Sugano S, Yamashita T, and Ikeda H (1995). Characterization of cDNA encoding mouse homolog of fission yeast dhp1+ gene: structural and functional conservation. Nucleic Acids Res. 23, 357–361. doi:10.1093/nar/23.3.357 7885830 PMC306683

[R264] Shuai K, and Warner JR (1991). A temperature sensitive mutant of *Saccharomyces cerevisiae* defective in pre-rRNA processing. Nucleic Acids Res. 19, 5059–5064. doi:10.1093/nar/19.18.5059 1923772 PMC328810

[R265] Sievers A, Beringer M, Rodnina MV, and Wolfenden R (2004). The ribosome as an entropy trap. Proc. Natl. Acad. Sci. U. S. A 101, 7897–7901. doi:10.1073/pnas.0402488101 15141076 PMC419528

[R266] Silva S, Homolka D, and Pillai RS (2017). Characterization of the mammalian RNA exonuclease 5/NEF-sp as a testis-specific nuclear 3’--> 5’ exoribonuclease. RNA 23, 1385–1392. doi:10.1261/rna.060723.117 28539487 PMC5558908

[R267] Sinclair DA, and Guarente L (1997). Extrachromosomal rDNA circles–a cause of aging in yeast. Cell 91, 1033–1042. doi:10.1016/s0092-8674(00)80493-6 9428525

[R268] Sloan KE, Mattijssen S, Lebaron S, Tollervey D, Pruijn GJ, and Watkins NJ (2013). Both endonucleolytic and exonucleolytic cleavage mediate ITS1 removal during human ribosomal RNA processing. J. Cell Biol 200, 577–588. doi:10.1083/jcb.201207131 23439679 PMC3587827

[R269] Sorensen PD, and Frederiksen S (1991). Characterization of human 5S rRNA genes. Nucleic Acids Res. 19, 4147–4151. doi:10.1093/nar/19.15.4147 1870970 PMC328554

[R270] Sorensen PD, Lomholt B, Frederiksen S, and Tommerup N (1991). Fine mapping of human 5S rRNA genes to chromosome 1q42.11–--q42.13. Cytogenet. Genome Res 57, 26–29. doi:10.1159/000133107 1855389

[R271] Srivastava L, Lapik YR, Wang M, and Pestov DG (2010). Mammalian DEAD box protein Ddx51 acts in 3’ end maturation of 28S rRNA by promoting the release of U8 snoRNA. Mol. Cell. Biol 30, 2947–2956. doi:10.1128/mcb.00226-10 20404093 PMC2876670

[R272] Srivastava R, Srivastava R, and Ahn SH (2016). The epigenetic pathways to ribosomal DNA silencing. Microbiol. Mol. Biol. Rev 80, 545–563. doi:10.1128/mmbr.00005-16 27250769 PMC4981667

[R273] Steffensen DM, Duffey P, and Prensky W (1974). Localisation of 5S ribosomal RNA genes on human chromosome 1. Nature 252, 741–743. doi:10.1038/252741a0 4437633

[R274] Steiner G, Kuechler E, and Barta A (1988). Photo-affinity labelling at the peptidyl transferase centre reveals two different positions for the A- and P-sites in domain V of 23S rRNA. EMBO J. 7, 3949–3955. doi:10.1002/j.1460-2075.1988.tb03281.x 3061810 PMC454989

[R275] Steitz TA, and Moore PB (2003). RNA, the first macromolecular catalyst: the ribosome is a ribozyme. Trends Biochem. Sci 28, 411–418. doi:10.1016/s0968-0004(03)00169-5 12932729

[R276] Stratmann S, Yones SA, Mayrhofer M, Norgren N, Skaftason A, Sun J, (2021). Genomic characterization of relapsed acute myeloid leukemia reveals novel putative therapeutic targets. Blood Adv. 5, 900–912. doi:10.1182/bloodadvances.2020003709 33560403 PMC7876890

[R277] Strohner R, Nemeth A, Jansa P, Hofmann-Rohrer U, Santoro R, Langst G, (2001). NoRC–a novel member of mammalian ISWI-containing chromatin remodeling machines. EMBO J. 20, 4892–4900. doi:10.1093/emboj/20.17.4892 11532953 PMC125270

[R278] Sulisalo T, Francomano CA, Sistonen P, Maher JF, McKusick VA, de la Chapelle A, (1994). High-resolution genetic mapping of the cartilage-hair hypoplasia (CHH) gene in amish and Finnish families. Genomics 20, 347–353. doi:10.1006/geno.1994.1187 8034306

[R279] Sulisalo T, Sistonen P, Hastbacka J, Wadelius C, Makitie O, de la Chapelle A, (1993). Cartilage-hair hypoplasia gene assigned to chromosome 9 by linkage analysis. Nat. Genet 3, 338–341. doi:10.1038/ng0493-338 7981754

[R280] Tague BW, and Gerbi SA (1984). Processing of the large rRNA precursor: two proposed categories of RNA-RNA interactions in eukaryotes. J. Mol. Evol 20, 362–367. doi:10.1007/bf02104742 6210374

[R281] Tanaka K, Kawai K, Kumahara Y, Ikenaga M, and Okada Y (1981). Genetic complementation groups in cockayne syndrome. Somat. Cell Genet 7, 445–455. doi:10.1007/bf01542989 7280930

[R282] Taoka M, Nobe Y, Yamaki Y, Sato K, Ishikawa H, Izumikawa K, (2018). Landscape of the complete RNA chemical modifications in the human 80S ribosome. Nucleic Acids Res. 46, 9289–9298. doi:10.1093/nar/gky811 30202881 PMC6182160

[R283] Taylor AB, Meyer B, Leal BZ, Kotter P, Schirf V, Demeler B, (2008). The crystal structure of Nep1 reveals an extended SPOUT-class methyltransferase fold and a pre-organized SAM-binding site. Nucleic Acids Res. 36, 1542–1554. doi:10.1093/nar/gkm1172 18208838 PMC2275143

[R284] Tiollais P, Galibert F, and Boiron M (1971). Evidence for the existence of several molecular species in the “45S fraction” of mammalian ribosomal precursor RNA. Proc. Natl. Acad. Sci. U. S. A 68, 1117–1120. doi:10.1073/pnas.68.6.1117 5288361 PMC389132

[R285] Tissenbaum HA, and Guarente L (2001). Increased dosage of a sir-2 gene extends lifespan in *Caenorhabditis elegans*. Nature 410, 227–230. doi:10.1038/35065638 11242085

[R286] Tone Y, and Toh-e A (2002). Nob1p is required for biogenesis of the 26S proteasome and degraded upon its maturation in *Saccharomyces cerevisiae*. Genes Dev. 16, 3142–3157. doi:10.1101/gad.1025602 12502737 PMC187499

[R287] Topper JN, and Clayton DA (1990). Characterization of human MRP/Th RNA and its nuclear gene: full length MRP/Th RNA is an active endoribonuclease when assembled as an RNP. Nucleic Acids Res. 18, 793–799. doi:10.1093/nar/18.4.793 1690392 PMC330329

[R288] Traub P, and Nomura M (1968). Structure and function of *E. coli* ribosomes. V. Reconstitution of functionally active 30S ribosomal particles from RNA and proteins. Proc. Natl. Acad. Sci. U. S. A 59, 777–784. doi:10.1073/pnas.59.3.777 4868216 PMC224743

[R289] Troelstra C, Odijk H, de Wit J, Westerveld A, Thompson LH, Bootsma D, (1990). Molecular cloning of the human DNA excision repair gene ERCC-6. Mol. Cell. Biol 10, 5806–5813. doi:10.1128/mcb.10.11.5806 2172786 PMC361360

[R290] Udem SA, and Warner JR (1973). The cytoplasmic maturation of a ribosomal precursor ribonucleic acid in yeast. J. Biol. Chem 248, 1412–1416. doi:10.1016/s0021-9258(19)44314-7 4568815

[R291] Ulhaq ZS, Nurputra DK, Soraya GV, Kurniawati S, Istifiani LA, Pamungkas SA, (2023). A systematic review on Treacher Collins syndrome: correlation between molecular genetic findings and clinical severity. Clin. Genet 103, 146–155. doi:10.1111/cge.14243 36203321

[R292] Umeda M, Ma J, Huang BJ, Hagiwara K, Westover T, Abdelhamed S, (2022). Integrated genomic analysis identifies UBTF tandem duplications as a recurrent lesion in pediatric acute myeloid leukemia. Blood Cancer Discov. 3, 194–207. doi:10.1158/2643-3230.bcd-21-0160 35176137 PMC9780084

[R293] Vakkilainen T, Kivipensas P, Kaitila I, de le Chapelle A, and Ridanpaa M (1999). Integrated high-resolution BAC, P1, and transcript map of the CHH region in chromosome 9p13. Genomics 59, 319–325. doi:10.1006/geno.1999.5883 10444333

[R294] Valdez BC, Henning D, So RB, Dixon J, and Dixon MJ (2004). The Treacher Collins syndrome (TCOF1) gene product is involved in ribosomal DNA gene transcription by interacting with upstream binding factor. Proc. Natl. Acad. Sci. U. S. A 101, 10709–10714. doi:10.1073/pnas.0402492101 15249688 PMC489999

[R295] van Hoof A, Lennertz P, and Parker R (2000). Three conserved members of the RNase D family have unique and overlapping functions in the processing of 5S U5, RNase MRP and RNase P RNAs in yeast. EMBO J. U4, 1357–1365. doi:10.1093/emboj/19.6.1357 PMC305676 10716935

[R296] van Tran N, Ernst FGM, Hawley BR, Zorbas C, Ulryck N, Hackert P, (2019). The human 18S rRNA m6A methyltransferase METTL5 is stabilized by TRMT112. Nucleic Acids Res. 47, 7719–7733. doi:10.1093/nar/gkz619 31328227 PMC6735865

[R297] Veiko NN, Ershova ES, Veiko RV, Umriukhin PE, Kurmyshev MV, Kostyuk GP, (2022). Mild cognitive impairment is associated with low copy number of ribosomal genes in the genomes of elderly people. Front. Genet 13, 967448. doi:10.3389/fgene.2022.967448 36199570 PMC9527325

[R298] Vulliamy T, Beswick R, Kirwan M, Marrone A, Digweed M, Walne A, (2008). Mutations in the telomerase component NHP2 cause the premature ageing syndrome dyskeratosis congenita. Proc. Natl. Acad. Sci. U. S. A 105, 8073–8078. doi:10.1073/pnas.0800042105 18523010 PMC2430361

[R299] Vulliamy T, Marrone A, Goldman F, Dearlove A, Bessler M, Mason PJ, (2001). The RNA component of telomerase is mutated in autosomal dominant dyskeratosis congenita. Nature 413, 432–435. doi:10.1038/35096585 11574891

[R300] Walker-Kopp N, Jackobel AJ, Pannafino GN, Morocho PA, Xu X, and Knutson BA (2017). Treacher Collins syndrome mutations in *Saccharomyces cerevisiae* destabilize RNA polymerase I and III complex integrity. Hum. Mol. Genet 26, 4290–4300. doi:10.1093/hmg/ddx317 28973381 PMC6251613

[R301] Walne AJ, Vulliamy T, Marrone A, Beswick R, Kirwan M, Masunari Y, (2007). Genetic heterogeneity in autosomal recessive dyskeratosis congenita with one subtype due to mutations in the telomerase-associated protein NOP10. Hum. Mol. Genet 16, 1619–1629. doi:10.1093/hmg/ddm111 17507419 PMC2882227

[R302] Wang M, and Lemos B (2017). Ribosomal DNA copy number amplification and loss in human cancers is linked to tumor genetic context, nucleolus activity, and proliferation. PLoS Genet. 13, e1006994. doi:10.1371/journal.pgen.1006994 28880866 PMC5605086

[R303] Wang M, and Pestov DG (2011). 5’-end surveillance by Xrn2 acts as a shared mechanism for mammalian pre-rRNA maturation and decay. Nucleic Acids Res. 39, 1811–1822. doi:10.1093/nar/gkq1050 21036871 PMC3061060

[R304] Warda AS, Freytag B, Haag S, Sloan KE, Gorlich D, and Bohnsack MT (2016). Effects of the Bowen-Conradi syndrome mutation in EMG1 on its nuclear import, stability and nucleolar recruitment. Hum. Mol. Genet 25, 5353–5364. doi:10.1093/hmg/ddw351 27798105 PMC5418833

[R305] Warner JR, Vilardell J, and Sohn JH (2001). Economics of ribosome biosynthesis. Cold Spring Harb. Symposia Quantitative Biol 66, 567–574. doi:10.1101/sqb.2001.66.567 12762058

[R306] Weber AM, Tuchweber B, Yousef I, Brochu P, Turgeon C, Gabbiani G, (1981). Severe familial cholestasis in North American Indian children: a clinical model of microfilament dysfunction? Gastroenterology 81, 653–662. doi:10.1016/0016-5085(81)90487-x 6894906

[R307] Wellauer PK, and Dawid IB (1979). Isolation and sequence organization of human ribosomal DNA. J. Mol. Biol 128, 289–303. doi:10.1016/0022-2836(79)90089-5 439136

[R308] Wells GR, Weichmann F, Colvin D, Sloan KE, Kudla G, Tollervey D, (2016). The PIN domain endonuclease Utp24 cleaves pre-ribosomal RNA at two coupled sites in yeast and humans. Nucleic Acids Res. 44, 5399–5409. doi:10.1093/nar/gkw213 27034467 PMC4914098

[R309] Wells GR, Weichmann F, Sloan KE, Colvin D, Watkins NJ, and Schneider C (2017). The ribosome biogenesis factor yUtp23/hUTP23 coordinates key interactions in the yeast and human pre-40S particle and hUTP23 contains an essential PIN domain. Nucleic Acids Res. 45, 4796–4809. doi:10.1093/nar/gkw1344 28082392 PMC5416842

[R310] Whitaker R, Faulkner S, Miyokawa R, Burhenn L, Henriksen M, Wood JG, (2013). Increased expression of Drosophila Sir2 extends life span in a dose-dependent manner. Aging (Albany NY) 5, 682–691. doi:10.18632/aging.100599 24036492 PMC3808700

[R311] Williams GT, and Farzaneh F (2012). Are snoRNAs and snoRNA host genes new players in cancer? Nat. Rev. Cancer 12, 84–88. doi:10.1038/nrc3195 22257949

[R312] Winokur ST, and Shiang R (1998). The Treacher Collins syndrome (TCOF1) gene product, treacle, is targeted to the nucleolus by signals in its C-terminus. Hum. Mol. Genet 7, 1947–1952. doi:10.1093/hmg/7.12.1947 9811939

[R313] Wise CA, Chiang LC, Paznekas WA, Sharma M, Musy MM, Ashley JA, (1997). TCOF1 gene encodes a putative nucleolar phosphoprotein that exhibits mutations in Treacher Collins Syndrome throughout its coding region. Proc. Natl. Acad. Sci. U. S. A 94, 3110–3115. doi:10.1073/pnas.94.7.3110 9096354 PMC20330

[R314] Wurm JP, Meyer B, Bahr U, Held M, Frolow O, Kotter P, (2010). The ribosome assembly factor Nep1 responsible for Bowen-Conradi syndrome is a pseudouridine-N1-specific methyltransferase. Nucleic Acids Res. 38, 2387–2398. doi:10.1093/nar/gkp1189 20047967 PMC2853112

[R315] Xie W, Ling T, Zhou Y, Feng W, Zhu Q, Stunnenberg HG, (2012). The chromatin remodeling complex NuRD establishes the poised state of rRNA genes characterized by bivalent histone modifications and altered nucleosome positions. Proc. Natl. Acad. Sci. U. S. A 109, 8161–8166. doi:10.1073/pnas.1201262109 22570494 PMC3361413

[R316] Xu H, and Hurley LH (2022). A first-in-class clinical G-quadruplex-targeting drug. The bench-to-bedside translation of the fluoroquinolone QQ58 to CX-5461 (Pidnarulex). Bioorg. Med. Chem. Lett 77, 129016. doi:10.1016/j.bmcl.2022.129016 36195286

[R317] Xue S, and Barna M (2012). Specialized ribosomes: a new frontier in gene regulation and organismal biology. Nat. Rev. Mol. Cell Biol 13, 355–369. doi:10.1038/nrm3359 22617470 PMC4039366

[R318] Xue Y, Bai X, Lee I, Kallstrom G, Ho J, Brown J, (2000). *Saccharomyces cerevisiae* RAI1 (YGL246c) is homologous to human DOM3Z and encodes a protein that binds the nuclear exoribonuclease Rat1p. Mol. Cell. Biol 20, 4006–4015. doi:10.1128/mcb.20.11.4006-4015.2000 10805743 PMC85771

[R319] Yamamoto M, and Seifart KH (1978). Heterogeneity in the 3’-terminal sequence of ribosomal 5S RNA synthesized by isolated HeLa cell nuclei *in vitro*. Biochemistry 17, 457–461. doi:10.1021/bi00596a013 620001

[R320] Yang XC, Purdy M, Marzluff WF, and Dominski Z (2006). Characterization of 3’hExo, a 3’ exonuclease specifically interacting with the 3’ end of histone mRNA. J. Biol. Chem 281, 30447–30454. doi:10.1074/jbc.m602947200 16912046

[R321] Yoon A, Peng G, Brandenburg Y, Zollo O, Xu W, Rego E, (2006). Impaired control of IRES-mediated translation in X-linked dyskeratosis congenita. Science 312, 902–906. doi:10.1126/science.1123835 16690864

[R322] Yu B, Mitchell GA, and Richter A (2005). Nucleolar localization of cirhin, the protein mutated in North American Indian childhood cirrhosis. Exp. Cell Res 311, 218–228. doi:10.1016/j.yexcr.2005.08.012 16225863

[R323] Yuan X, Feng W, Imhof A, Grummt I, and Zhou Y (2007). Activation of RNA polymerase I transcription by cockayne syndrome group B protein and histone methyltransferase G9a. Mol. Cell 27, 585–595. doi:10.1016/j.molcel.2007.06.021 17707230

[R324] Yuan Y, Singh R, and Reddy R (1989). Rat nucleolar 7–2 RNA is homologous to mouse mitochondrial RNase mitochondrial RNA-processing RNA. J. Biol. Chem 264, 14835–14839. doi:10.1016/s0021-9258(18)63776-7 2475491

[R325] Yusupov MM, Yusupova GZ, Baucom A, Lieberman K, Earnest TN, Cate JH, (2001). Crystal structure of the ribosome at 5.5 A resolution. Science 292, 883–896. doi:10.1126/science.1060089 11283358

[R326] Zafiropoulos A, Tsentelierou E, Linardakis M, Kafatos A, and Spandidos DA (2005). Preferential loss of 5S and 28S rDNA genes in human adipose tissue during ageing. Int. J. Biochem. Cell Biol 37, 409–415. doi:10.1016/j.biocel.2004.07.007 15474985

[R327] Zhang T, Chen P, Li W, Sha S, Wang Y, Yuan Z, (2019). Cognitive deficits in mice lacking Nsun5, a cytosine-5 RNA methyltransferase, with impairment of oligodendrocyte precursor cells. Glia 67, 688–702. doi:10.1002/glia.23565 30485550

[R328] Zhao J, Yuan X, Frodin M, and Grummt I (2003). ERK-dependent phosphorylation of the transcription initiation factor TIF-IA is required for RNA polymerase I transcription and cell growth. Mol. Cell 11, 405–413. doi:10.1016/s1097-2765(03)00036-4 12620228

[R329] Zhao Y, Rai J, and Li H (2023). Regulation of translation by ribosomal RNA pseudouridylation. Sci. Adv 9, eadg8190. doi:10.1126/sciadv.adg8190 37595043 PMC10438446

[R330] Zhou F, Aroua N, Liu Y, Rohde C, Cheng J, Wirth AK, (2023). A dynamic rRNA ribomethylome drives stemness in acute myeloid leukemia. Cancer Discov. 13, 332–347. doi:10.1158/2159-8290.cd-22-0210 36259929 PMC9900322

[R331] Zhou F, Liu Y, Rohde C, Pauli C, Gerloff D, Kohn M, (2017). AML1-ETO requires enhanced C/D box snoRNA/RNP formation to induce self-renewal and leukaemia. Nat. Cell Biol 19, 844–855. doi:10.1038/ncb3563 28650479

[R332] Zillner K, Komatsu J, Filarsky K, Kalepu R, Bensimon A, and Nemeth A (2015). Active human nucleolar organizer regions are interspersed with inactive rDNA repeats in normal and tumor cells. Epigenomics 7, 363–378. doi:10.2217/epi.14.93 26077426

[R333] Zomerdijk JC, Beckmann H, Comai L, and Tjian R (1994). Assembly of transcriptionally active RNA polymerase I initiation factor SL1 from recombinant subunits. Science 266, 2015–2018. doi:10.1126/science.7801130 7801130

